# Molecular Mechanisms and Therapeutic Targeting of the STAT3 Signaling Axis in Vascular Smooth Muscle Cell Phenotypic Switching and Vascular Remodeling

**DOI:** 10.3390/cells15171624

**Published:** 2026-09-07

**Authors:** Tingxuan Zheng, Haomin Li, Long Yao, Yiqian Zhang, Lingran Feng, Dongmei Yang

**Affiliations:** 1 School of Integrated Chinese and Western Medicine, Hunan University of Chinese Medicine, Changsha 410208, China; 202307010431@stu.hnucm.edu.cn (T.Z.); 202307010735@stu.hnucm.edu.cn (L.F.); 2 Medical School, Hunan University of Chinese Medicine, Changsha 410208, China; resplendent_hawk@stu.hnucm.edu.cn (H.L.); yaolong@stu.hnucm.edu.cn (L.Y.); 202308010232@stu.hnucm.edu.cn (Y.Z.)

**Keywords:** STAT3, VSMCs, cardiovascular diseases, vascular remodeling, signaling pathways, targeted therapy, clinical translation

## Abstract

Vascular smooth muscle cells (VSMCs) exhibit remarkable phenotypic plasticity, dynamically transitioning from a quiescent contractile state to a dedifferentiated synthetic phenotype that constitutes the fundamental cytological driver of pathological vascular remodeling in cardiovascular diseases (CVDs) including atherosclerosis, vascular restenosis, aortic dissection (AD), and vascular calcification (VC). Signal transducer and activator of transcription 3 (STAT3) operates as a master transcriptional and functional convergence node, integrating diverse upstream biochemical stimuli, neurohumoral factors, and biomechanical stressors to govern downstream gene regulatory networks. Aberrant STAT3 activation orchestrates VSMC phenotypic modulation, excessive proliferation, directional migration, programmed cell death involving apoptosis resistance and pyroptosis initiation, extracellular matrix (ECM) reorganization, and glycolytic metabolic reprogramming via canonical nuclear transcription and non-canonical subcellular actions. Here, we systematically delineate the modular structural organization, post-translational modifications, and negative regulatory feedback mechanisms of STAT3 in shaping VSMC functionality. Furthermore, we synthesize recent advances in pharmacological interventions by comprehensively categorizing therapeutic modalities into direct structural-domain inhibitors, upstream kinase-targeted indirect agents, bioactive natural products, and emerging clinical-stage translational candidates. Finally, we critically address translational hurdles regarding on-target systemic toxicities, and highlight site-specific vascular delivery systems alongside localized drug-eluting vascular devices to advance precision STAT3-targeted cardiovascular therapeutics.

## 1. Introduction

Cardiovascular disease (CVD), an overarching descriptor for disorders affecting the heart and circulatory system, remains a leading cause of global morbidity and mortality [1]. This cluster of pathologies accounts for approximately 17.9 million fatalities annually, constituting 31% of all deaths worldwide [2]. Despite a steady decline in age-standardized incidence and mortality rates over the past four decades, CVD continues to impose an immense burden on public healthcare systems and presents profound socioeconomic challenges [3]. Consequently, an in-depth elucidation of the core cytological substrates and molecular regulatory networks driving CVD progression is of paramount importance for the prevention and therapeutic management of these disorders.

Vascular smooth muscle cells (VSMCs) constitute the primary cellular component of the tunica media, playing an indispensable role in maintaining vascular structural and functional homeostasis [4]. Under physiological conditions, VSMCs maintain basal vascular tone and regulate vessel caliber [5,6]. By expressing contractile proteins and generating mechanical force, VSMCs modulate vasomotor states to regulate blood flow distribution and blood pressure [7,8], while actively maintaining extracellular matrix (ECM) homeostasis. However, VSMCs are not terminally differentiated cells; instead, they exhibit remarkable phenotypic plasticity in response to diverse microenvironmental cues [9,10,11]. Upon exposure to pathological insults such as vascular injury, pro-inflammatory cytokines, or mechanical stress, VSMCs undergo a transition from a highly differentiated contractile phenotype to a dedifferentiated synthetic phenotype [12,13,14]. This phenotypic switching is characterized by markedly augmented capacities for cell proliferation, migration, ECM synthesis, and metabolic reprogramming, serving as a common cytopathological foundation for a wide spectrum of CVDs, including atherosclerosis, restenosis after percutaneous coronary intervention, and aneurysms [15,16,17]. Accumulating evidence indicates that VSMCs phenotypic modulation is dynamically governed by a sophisticated intracellular signaling network, within which signal transducer and activator of transcription 3 (STAT3) serves as a critically pivotal player [18,19].

**Figure 1:**
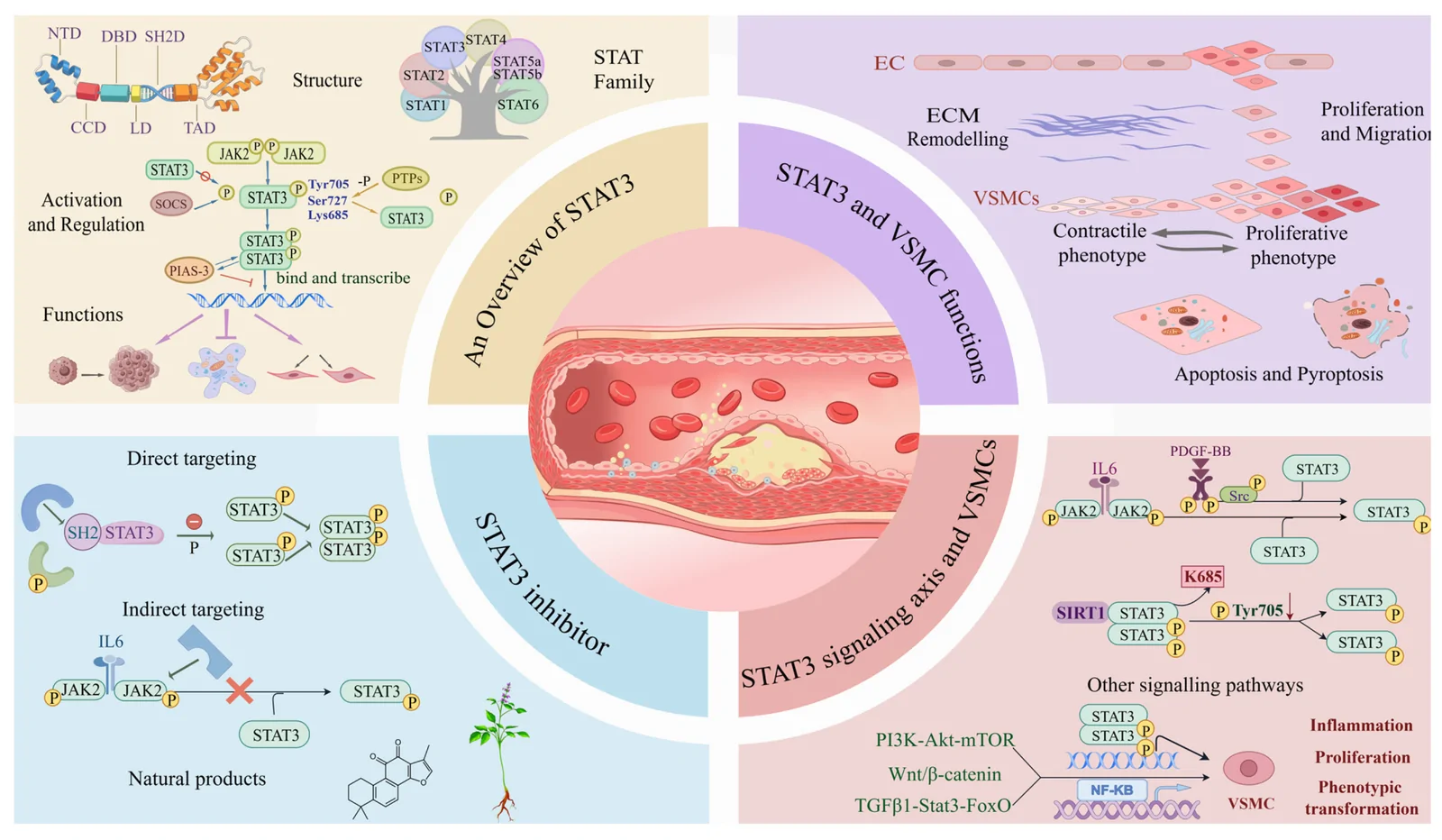
Schematic overview of the STAT3 signaling axis in VSMCs functional remodeling and therapeutic targeting. An Overview of STAT3: Structural domains of STAT3 (NTD to TAD) and its post-translational activation (phosphorylation and acetylation), strictly regulated by upstream kinases (JAK2) and negative feedback loops (SOCS, PIAS, and PTPs). STAT3 and VSMCs Functions: Pathological activation of STAT3 driving VSMCs phenotypic switching from the contractile to synthetic phenotype, subsequently orchestrating aberrant proliferation, migration, ECM remodeling, apoptosis, and pyroptosis. STAT3 Signaling Axis and Cross-talk: Molecular networks intersecting with STAT3, including the classical IL-6/JAK2 and PDGF-BB/Src cascades, SIRT1-mediated deacetylation, and reciprocal cross-talk with parallel pathways (PI3K-Akt-mTOR, Wnt/β-catenin, and TGF-β1/Stat3-FoxO). STAT3 Inhibitors: Stratification of therapeutic strategies counteracting hyperactive STAT3, classified into direct targeting (SH2 domain dimerization inhibitors), indirect targeting (upstream kinase inhibitors), and multi-target bioactive natural products. Note: Solid arrows indicate activation or promotion; T-shaped bars represent inhibition; double-headed arrows indicate mutual phenotypic conversion. Created in Figdraw.

As a master node within the VSMCs regulatory landscape, the activation state of STAT3 is a decisive determinant of VSMCs phenotypic switching and functional dysregulation [20,21,22,23]. STAT3 signaling orchestrates multiple downstream biological processes—including proliferation, migration, secretory profiles, and metabolic reprogramming—thereby fundamentally dictating vascular homeostasis and remodeling [24,25]. Notably, the deleterious effects of STAT3-mediated VSMCs dysfunction manifest with distinct pathological traits depending on the specific disease context. Specifically, STAT3 drives aberrant VSMC proliferation and synthetic switching in atherosclerosis and restenosis [22,26,27], regulates apoptosis and matrix turnover in aneurysms and aortic dissections (ADs) [28,29,30], and governs osteogenic transdifferentiation in vascular calcification [31,32]. Collectively, these insights establish STAT3 as a central molecular hub that integrates diverse pathological stimuli, elicits specific aberrant VSMCs phenotypes, and ultimately culminates in divergent cardiovascular disorders. Consequently, therapeutic targeting of the STAT3 signaling axis has emerged as a promising strategy for cardiovascular intervention. Based on their distinct modes of action, STAT3 inhibitors are primarily categorized into: direct small-molecule compounds targeting specific functional domains, indirect agents that intercept upstream kinase cascades (e.g., JAKs) or cell-surface receptors, and bioactive natural products with verified STAT3-inhibitory properties. Here, we provide an integrative conceptual framework focusing on the multifaceted roles of STAT3 in modulating VSMCs cellular physiology and pathology. Furthermore, we comprehensively summarize current pharmacological strategies targeting the STAT3 axis, aiming to establish a robust theoretical foundation for the development of innovative therapeutic approaches for CVDs(Figure 1).

**Literature Search Strategy and Selection Criteria:** To ensure a comprehensive, rigorous, and up-to-date synthesis of evidence, the literature searches were systematically performed across PubMed, Web of Science, and Google Scholar databases up to June 2026. The search strategy incorporated key terms and Medical Subject Headings (MeSH): (“STAT3” OR “signal transducer and activator of transcription 3”) AND (“vascular smooth muscle cell” OR “VSMC” OR “phenotypic switching” OR “vascular remodeling”) AND (“atherosclerosis” OR “aortic dissection” OR “vascular calcification” OR “inhibitor” OR “targeted therapy”). Priority was given to peer-reviewed original research articles and high-impact reviews published within the past three to five years, particularly those offering definitive mechanistic insights into VSMC plasticity and vascular pathology. Non-peer-reviewed preprints and studies lacking rigorous mechanistic validation were excluded.

## 2. Structure, Activation Regulation, and Function of STAT3

Signal transducers and activators of transcription (STATs) represent a highly conserved family of transcription factors that are widely involved in critical biological processes, including cell proliferation, differentiation, apoptosis, and immune responses [33]. In mammals, the STAT family comprises seven members, namely STAT1, STAT2, STAT3, STAT4, STAT5a, STAT5b, and STAT6 [34]. Among these seven members, STAT3 has become one of the most comprehensively investigated and biologically complex molecules due to its profound functional diversity and widespread tissue distribution. Accumulating evidence in recent years indicates that the aberrant activation of the STAT3 signaling pathway is closely linked to the pathogenesis and progression of various CVDs [35,36,37].

### 2.1. Structural Domains of STAT3

**Figure 2:**
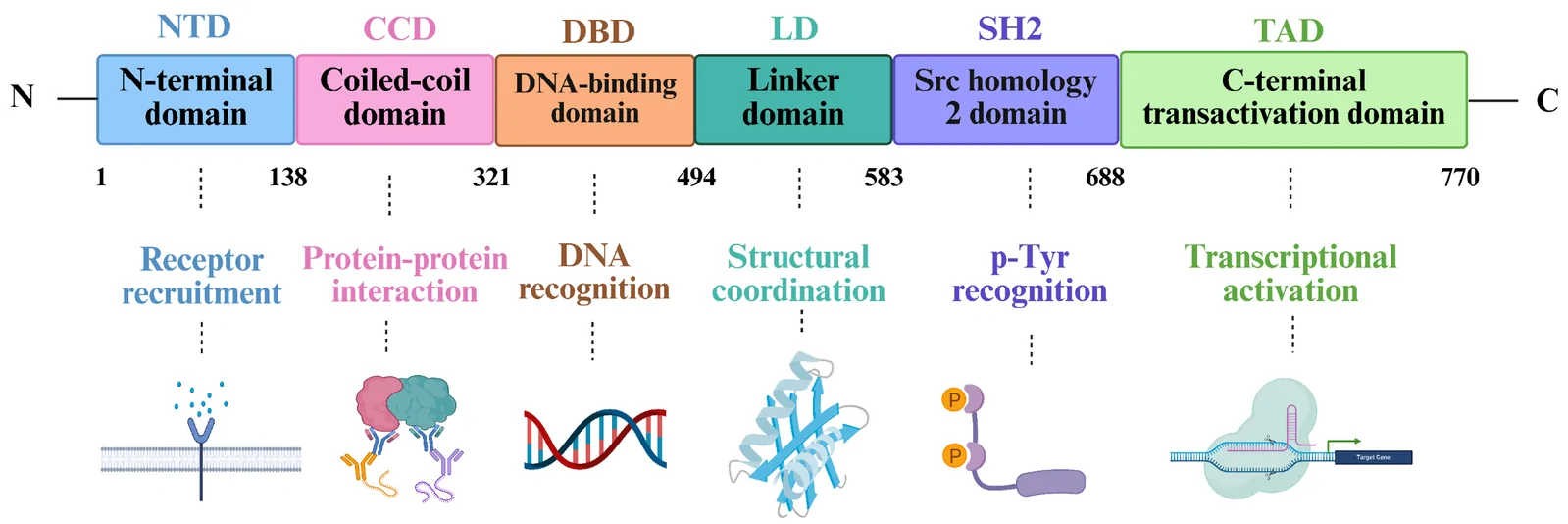
Structural organization and functional domains of STAT3. STAT3 is a modular transcription factor composed of six conserved domains arranged from the N-terminus to the C-terminus: the NTD, CCD, DBD, LD, SH2D, and TAD. The NTD facilitates receptor recruitment and contributes to STAT3 phosphorylation, dimerization, and nuclear translocation. The CCD mediates protein–protein interactions and may participate in DNA-binding specificity. The DBD recognizes specific DNA elements within target gene promoters, whereas the LD maintains the structural coordination between the DBD and SH2D. The SH2D is essential for phosphotyrosine recognition and STAT3 homodimer formation. The TAD contains the critical phosphorylation sites Tyr705 and Ser727 and recruits transcriptional co-activators, such as p300/CBP, to enhance transcriptional activation of downstream target genes. The figure was created in BioRender.

STAT3 possesses an exquisite and modular structural organization, enabling it to effectively respond to extracellular signals and mediate gene transcriptional regulation [38]. The STAT3 protein is primarily composed of six functional domains arranged sequentially from the N-terminus to the C-terminus: NTD, CCD, DBD, LD, SH2, and TAD, which cooperatively ensure the normal execution of STAT3 functions [39] (Figure 2). N-terminal domain (NTD, aa 1–138): Located at the N-terminus, the conserved NTD facilitates receptor recruitment, phosphorylation, dimerization, and nuclear translocation of STAT3 dimers [40].

Coiled-coil domain (CCD, aa 138–321): Composed of α-helices, the CCD derives its name from its coiled-coil conformation and is situated adjacent to the NTD [41]. The CCD primarily mediates interactions between STAT3 and other proteins; Dumoutier et al. discovered that the C-terminus of the IL-22 receptor can recruit the STAT3 CCD in a tyrosine-independent manner, demonstrating that tyrosine phosphorylation of IL-22R is not mandatory for STAT3 activation, thereby providing new insights into the modes of STAT3 activation [42]. Additionally, the CCD may participate in the specificity of DNA binding and contribute to STAT3 oligomerization and its interaction with specific DNA sequences, thus regulating gene expression [43]. DNA-binding domain (DBD, aa 321–494): The DBD is a highly conserved region capable of specifically recognizing and binding to DNA, positioned immediately after the CCD [41]. The DBD is paramount for STAT3 to exert its function as a transcription factor, allowing it to recognize and bind to specific DNA sequences within target gene promoter regions [39]. Studies have shown that the DBD of STAT3 mediates its interaction with NF-κB p65, thereby suppressing the transcription of inducible nitric oxide synthase (iNOS) [44].

Linker domain (LD, aa 494–583): Spanning residues 494 to 583, the LD connects the DBD and SH2 domains [14], serving as a structural scaffold to coordinate domain conformations and overall protein flexibility [39,41]. SH2 domain (Src homology 2 domain, aa 583–688): The SH2 domain is a highly conserved protein module situated after the LD, spanning amino acid positions 583 to 688 [39]. Furthermore, the SH2 domain can interact with the phosphorylated Tyr705 site of another STAT3 molecule to form a stable trans-interaction, which serves as the structural foundation for homodimerization [45].

C-terminal transactivation domain (TAD, aa 688–770): The C-terminal TAD harbors key phosphorylation sites (Tyr705 and Ser727) [46] and recruits co-activators such as p300/CBP to drive gene transcription [47]. Ser727 phosphorylation enhances p300/CBP recruitment and chromatin remodeling to facilitate downstream transcription [48].

### 2.2. Activation and Regulatory Mechanisms of STAT3

**Figure 3:**
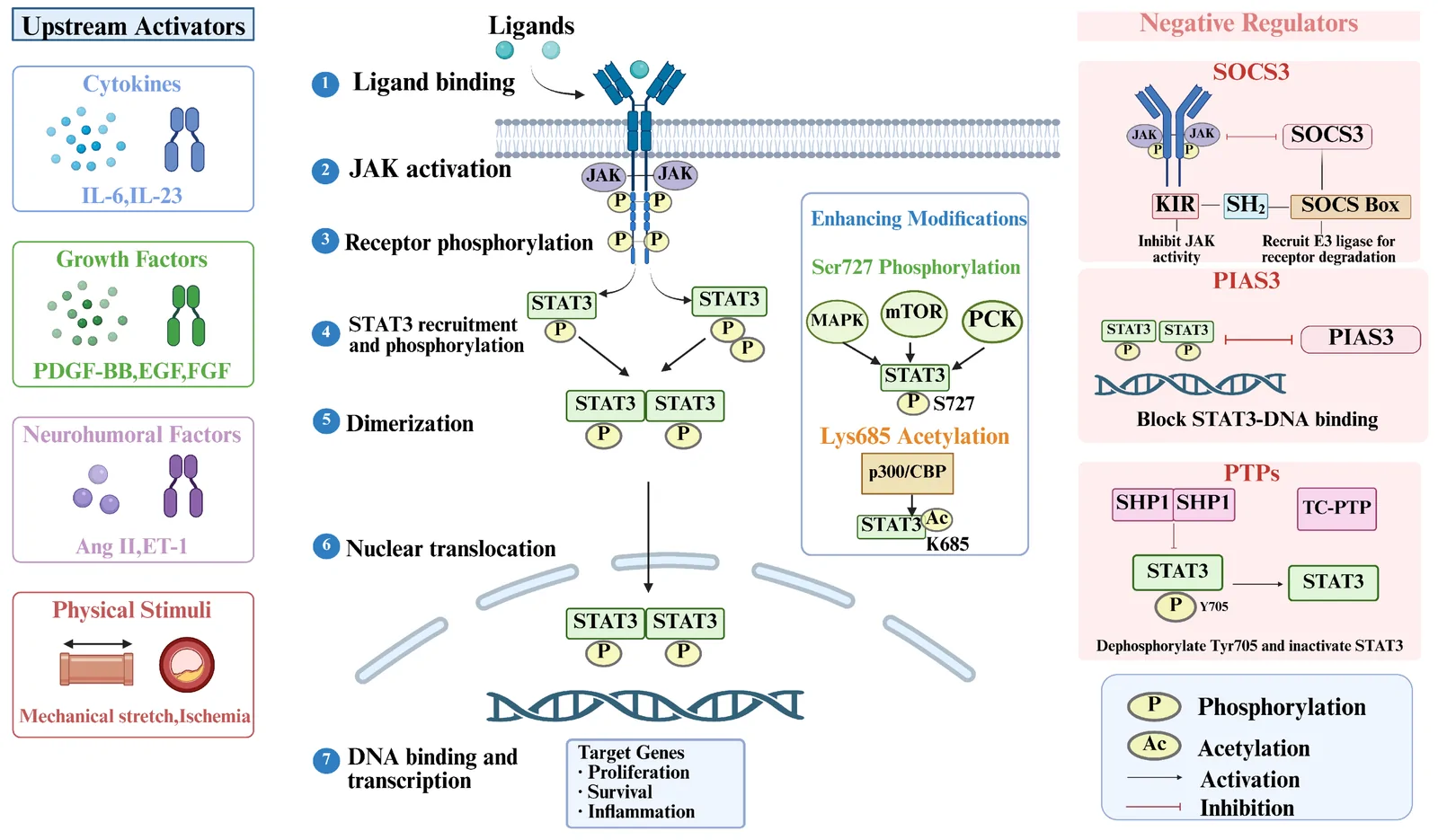
Activation and regulatory mechanisms of STAT3 signaling. STAT3 signaling can be activated by a wide range of extracellular stimuli, including cytokines (e.g., IL-6, IL-11, and IL-23), growth factors (e.g., PDGF-BB, EGF, and FGF), neurohumoral mediators (e.g., AngII and endothelin-1), as well as physical stressors such as mechanical stretch and ischemia. Upon ligand binding, receptor-associated Janus kinases (JAKs) are activated and induce receptor phosphorylation, creating docking sites for STAT3 recruitment. STAT3 is subsequently phosphorylated at Tyr705, leading to homodimerization through reciprocal SH2 domain interactions and nuclear translocation. In the nucleus, activated STAT3 binds to specific DNA response elements and regulates the transcription of genes involved in cellular proliferation, survival, inflammation, and other biological processes. In addition to canonical Tyr705 phosphorylation, STAT3 activity can be further enhanced by non-canonical post-translational modifications, including Ser727 phosphorylation mediated by MAPKs, mTOR, and PKC, and Lys685 acetylation mediated by p300/CBP. These modifications modulate STAT3 transcriptional activity, protein stability, and signaling output. STAT3 signaling is tightly controlled by multiple negative regulatory mechanisms. SOCS3 suppresses JAK activity and promotes feedback inhibition of cytokine signaling, PIAS3 inhibits STAT3-dependent transcription by blocking STAT3–DNA interactions, and PTPs, such as SHP1, SHP2, and TC-PTP, terminate signaling through dephosphorylation of activated STAT3. Collectively, these regulatory mechanisms ensure precise control of STAT3 signaling under physiological and pathological conditions. The figure was created in BioRender.

The activation and regulation of STAT3 represent a complex and precise biological process that is vital for normal cellular functions. The activation of STAT3 is primarily achieved through JAK kinase-mediated Tyr705 phosphorylation and dimerization, while modifications such as Ser727 phosphorylation and Lys685 acetylation cooperatively enhance its transcriptional activity [49,50,51,52]; Conversely, its regulatory mechanisms are predominantly associated with three major negative feedback families: SOCS, PIAS, and PTPs [53,54,55] (Figure 3).

#### 2.2.1. Activation Mechanisms of STAT3

Accumulating evidence indicates that a diverse array of upstream signals can activate STAT3. These mainly include cytokines (such as IL-6 and IL-23) and growth factors, with the latter represented by platelet-derived growth factor (PDGF), epidermal growth factor (EGF), and fibroblast growth factor (FGF). Additionally, neurohumoral factors (such as AngII and endothelin-1) and physical stimuli (such as mechanical stretch stress and ischemia) also serve as upstream activators [56,57]. All of these stimuli can activate STAT3 through a common signaling hub, the Janus kinases (JAKs) [58,59].

The JAK-STAT3 signaling pathway represents the most predominant route for STAT3 activation. JAKs belong to the family of non-receptor tyrosine kinases, characterized by their lack of direct integration into the plasma membrane; instead, they constitutively pre-associate with the intracellular juxtamembrane regions of cytokine or growth factor receptors (hereinafter referred to as “signaling receptors”) [60]. Upon the binding of upstream ligands (such as IL-6 or PDGF) to their specific cell-surface receptors, this engagement induces the dimerization of two receptor subunits—meaning two signaling receptor monomers approach each other to form a stable homodimer or heterodimer. Dimerization of the signaling receptors spatially brings the associated JAKs, which were originally independently bound to the intracellular segments of the two receptor chains, into close proximity, enabling molecular interactions between them. Under these close-contact conditions, one JAK kinase molecule catalyzes the phosphorylation of a specific tyrosine residue on the adjacent JAK kinase molecule—a process termed trans-phosphorylation—which activates the JAKs and endows them with catalytic activity. The activated JAKs subsequently utilize their catalytic sites to phosphorylate specific tyrosine residues on the intracellular domains of the signaling receptors, thereby generating phosphotyrosine residues along these segments. These phosphotyrosine residues serve as high-affinity molecular docking sites, providing a precise binding platform for the downstream signaling molecule STAT3. At this juncture, cytosolic resting STAT3 monomers are precisely recognized and recruited to the signaling receptor complex via their SH2 domains, where they undergo phosphorylation at the tyrosine 705 residue (Tyr705) catalyzed by the associated JAK kinase [61]. This phosphorylation event constitutes the landmark event of STAT3 activation and is a prerequisite for its transcriptional activity. The phosphorylated Tyr705 on STAT3 immediately serves as a binding site for the SH2 domain of another STAT3 molecule; through reciprocal SH2-Tyr705 trans-interactions, two STAT3 monomers form a stable antiparallel homodimer. Following dimerization, the nuclear localization signal of the STAT3 dimer is exposed, allowing it to be highly efficiently translocated from the cytoplasm into the nucleus via the mediation of the nuclear transport receptor system. Upon entry into the nucleus, the STAT3 dimer binds to specific DNA response elements within the promoter regions of target genes (such as 5′-TTCNNNGAA-3′, termed the GAS element) to initiate the transcription of downstream target genes, thereby regulating a series of crucial biological processes including cell proliferation, apoptosis, and inflammatory responses [62,63,64].

In addition to the Tyr705 site, phosphorylation at the serine 727 site (Ser727) represents a critical cooperative modification event during STAT3 activation. While Tyr705 phosphorylation confers baseline DNA binding, maximal STAT3 transactivation requires Ser727 phosphorylation catalyzed by MAPKs, mTOR, or PKCδ to optimize co-activator recruitment [65]. Notably, Ser727 phosphorylation does not merely passively assist Tyr705 phosphorylation; it can independently regulate STAT3 activity through various upstream kinase networks. For instance, studies have revealed that in endothelial cells, protease-activated receptor 1 (PAR-1) signaling can lift the inhibition of glycogen synthase kinase 3α/β (GSK-3α/β) by blocking AKT, allowing GSK-3α/β to directly phosphorylate STAT3 at the Ser727 residue, thereby achieving STAT3 activation independent of Tyr705 phosphorylation [66].

Acetylation at the lysine 685 (Lys685) site within the STAT3 domain likewise plays an indispensable role in STAT3 activation. Situated within the C-terminal transactivation domain of STAT3, this site serves as the primary target for acetylation modifications mediated by the transcriptional co-activator p300/CREB-binding protein (CBP) [67]. Early studies demonstrated that p300/CBP-catalyzed acetylation of STAT3 at Lys685 significantly enhances its sequence-specific DNA-binding capacity and transactivation potency; furthermore, suppressing histone deacetylase activity further increases the nuclear retention of STAT3, indicating an independent regulatory role for acetylation in the process of STAT3 activation [67]. Further mechanistic investigations have indicated that acetylation at the Lys685 residue is paramount for the formation of stable STAT3 dimers, and this dimeric stability serves as the structural foundation for DNA binding and transcriptional regulation in response to cytokine stimulation [68].

#### 2.2.2. Regulatory Mechanisms of STAT3

The activity of STAT3 is finely tuned by a sophisticated intracellular network to ensure its normal function under physiological conditions and prevent overactivation under pathological circumstances. Currently recognized negative regulatory mechanisms of STAT3 predominantly involve three major families: suppressors of cytokine signaling (SOCS), protein inhibitors of activated STAT (PIAS), and protein tyrosine phosphatases (PTPs) [53,69,70].

Among numerous negative regulators, SOCS3 represents the most comprehensively investigated and functionally central negative feedback modulator within the STAT3 signaling cascade [71,72,73]. The expression of SOCS3 is directly driven by the transcriptional activation of STAT3, as its promoter region harbors two functional STAT3-binding elements; consequently, when STAT3 is activated to initiate the transcription of downstream target genes, SOCS3 induction is synchronously triggered [74]. Newly synthesized SOCS3 protein feedback-inhibits further STAT3 activation, thereby establishing a classical negative feedback regulatory loop. Specifically, the C-terminus of the SOCS3 protein contains an SH2 domain capable of specifically recognizing and binding to phosphorylated tyrosine residues within the activated receptor-JAK signaling complex, including the specific sites on the intracellular segments of cytokine receptors phosphorylated by JAKs as well as the phosphorylated tyrosine residues within the activation loop of JAKs themselves [75,76]. Following the precise targeting achieved by the SH2 domain, the N-terminal kinase inhibitory region (KIR) of SOCS3 directly inserts into the active center of the JH1 catalytic domain of JAKs, blocking the interaction between the kinase and its substrates via steric hindrance, thereby terminating the JAK-mediated STAT3 tyrosine phosphorylation signal at its source. Furthermore, the C-terminus of SOCS3 harbors a SOCS box that can recruit molecules such as Elongin B/C and Cullin 5 to assemble an E3 ubiquitin ligase complex, which mediates the degradation of cytokine receptors and thoroughly intercepts the activation of the JAK-STAT3 pathway at the signal initiation stage [77].

The PIAS family constitutes another class of negative regulators targeting STAT3 parallel to the SOCS family. Initially identified via yeast two-hybrid screening, members of this family comprise seven proteins encoded by four genes that are highly conserved across eukaryotic and mammalian cells, namely PIAS1, PIASxα, PIASxβ, PIAS3, PIAS3L, PIASy, and PIASyE6 (also designated as PIAS4) [78]. While all PIAS family members can negatively regulate STAT signaling pathways, PIAS3 exhibits the most intimate regulatory relationship with STAT3. PIAS3 was first identified in 1997 in mouse and human cell lines as an inhibitor of interferon-α-induced STAT3 transcriptional regulation [78]. Distinct from the mechanism of SOCS3, which represses upstream JAK kinase activity, PIAS3 does not block upstream signal transduction; instead, it physically binds to the DBD of the STAT3 dimer within the nucleus, specifically blocking the engagement of the STAT3 dimer with DNA response elements in the target gene promoters, thereby terminating STAT3-mediated gene expression at the transcriptional level [53,79].

The PTP family represents the third major class of molecules responsible for the negative regulation of STAT3 signaling, operating through the most direct mechanism: catalyzing the dephosphorylation of STAT3 at the Tyr705 site to restore it from an active phosphorylated state back to an inactive resting state [53,80,81]. This family encompasses seven PTP members with verified capacities to dephosphorylate STAT3, including receptor-type PTP D (PTPRD), receptor-type PTP T (PTPRT), receptor-type PTP K (PTPRK), Src homology 2 domain-containing protein tyrosine phosphatase 1 (SHP1) and 2 (SHP2), MEG2/PTP non-receptor type 9 (PTPN9), and T-cell PTP (TC-PTP)/PTP non-receptor type 2 (PTPN2) [59]. Among these family members, TC-PTP, SHP1, and SHP2 play the most prominent cooperative roles in modulating STAT3 dephosphorylation [82]. Within the cell, the nuclear isoform TC45 (the nuclear localization variant of TC-PTP) acts on STAT3 within the nucleus to induce its dephosphorylation [83,84], whereas SHP1 and SHP2 predominantly operate within the cytoplasm [52,85]. The orchestrated synergy among these three phosphatases collectively fine-tunes the global phosphorylation levels of STAT3.

### 2.3. Basic Functions of STAT3

STAT3 orchestrates cellular signal transduction through two complementary functional modes: canonical and non-canonical pathways. The canonical functions of STAT3 conventionally refer to its classic role as a nuclear transcription factor, predominantly initiated by cytokine- or growth factor-induced Tyr705 phosphorylation, SH2 domain-mediated dimerization, nuclear translocation, and sequence-specific DNA binding to target gene promoters. In contrast, non-canonical functions denote transcription-independent or extranuclear activities, such as mitochondrial energy regulation (mitoSTAT3), endoplasmic reticulum calcium homeostasis (ERSTAT3), and cytoplasmic protein–protein interactions.

#### 2.3.1. Canonical Functions of STAT3

As a classical transcription factor, the canonical activity of STAT3 predominantly relies on Tyr705 phosphorylation, which drives homodimerization, nuclear translocation, and direct engagement with sequence-specific response elements (such as GAS motifs) within target gene promoters [86,87,88]. Through this transcriptional machinery, canonical STAT3 coordinates a broad spectrum of downstream biological programs, primarily governing cell proliferation, anti-apoptosis, differentiation and phenotypic switching, and metabolic reprogramming.

The core mechanism by which STAT3 regulates cell proliferation lies in its direct activation of the transcriptional expression of key cell cycle regulators, such as the proto-oncogene c-Myc, Cyclin D1, and proliferating cell nuclear antigen (PCNA), thereby driving the orderly progression of the cell cycle [89]. In the transcriptional regulation of c-Myc, STAT3 recruits cofactors through its SH2 domain to form a complex that binds to the promoter region of the c-Myc gene, activating its transcription [90,91]. As a master transcription factor, c-Myc drives the transition of the cell cycle from the G1 to S phase by upregulating the expression of various G1/S phase regulators [92,93,94]. In the transcriptional regulation of Cyclin D1, activated STAT3 can directly bind to specific binding sites within the promoter region of the Cyclin D1 gene, activating Cyclin D1 transcription [95,96]. Cyclin D1 forms an active complex with CDK4 or CDK6, which phosphorylates the retinoblastoma protein to release E2F transcription factors, thereby initiating the transcription of S phase-related genes [97,98]. PCNA, serving as an auxiliary protein for DNA polymeraseδ, is a crucial factor indispensable for DNA replication and cell division, and its expression is likewise transcriptionally regulated by STAT3 [99]. Studies have shown that activation of the STAT3 signaling pathway significantly upregulates PCNA expression, thereby participating in the regulation of cell proliferation [100,101]. The transcriptional activation of these target genes by STAT3 is not an isolated event; c-Myc upregulates the expression of multiple G1/S regulators including Cyclin D1, forming a synergistic effect with the direct transcriptional activation of Cyclin D1 by STAT3, collectively ensuring the orderly advancement of the cell cycle.

The core mechanism of STAT3 participation in cell anti-apoptosis also relies on its direct binding to the promoter regions of anti-apoptotic protein genes and transcriptional activation of their expression, thereby blocking the transmission of apoptotic signals at multiple levels. Research indicates that STAT3 can simultaneously upregulate the transcription of Bcl-2 family anti-apoptotic members Bcl-2, Bcl-xL, and Mcl-1, which maintain membrane potential stability on the mitochondrial membrane and inhibit cytochrome c release, thereby exerting inhibitory effects upstream of the intrinsic apoptotic pathway [102,103]. Conversely, inhibiting STAT3 activation with AG490 or blocking STAT3 function with dominant-negative STAT3 significantly decreases the mRNA and protein levels of Bcl-2, Mcl-1, and c-IAP2, while increasing caspase-3 activity and PARP cleavage fragments, thereby inducing cell apoptosis [104,105]. Additionally, STAT3 can transcriptionally activate IAP family members Survivin and c-IAP2 to directly inhibit the activities of caspase-3, caspase-7, and caspase-9 during the apoptosis execution phase, blocking further transmission of apoptotic signals [106]. Through these mechanisms, STAT3 plays an irreplaceable and central role as a key anti-apoptotic transcription factor in regulating cell survival.

The regulation of cell differentiation and phenotypic switching by STAT3 is a core component of its canonical functions. Initially identified as an acute-phase response factor, STAT3 precisely regulates the transition of cells from a differentiated state to a dedifferentiated state by directly binding to the promoter regions of differentiation-related target genes, playing a key role in the development and homeostasis maintenance of various tissues and cell types [107]. The regulation of cell differentiation and phenotypic switching by STAT3 is achieved through multiple mechanisms. Specifically, STAT3 can directly bind to STAT-binding elements in the promoters of differentiation-related target genes to suppress the transcription of differentiation marker genes, thereby maintaining cells in a dedifferentiated state. Furthermore, STAT3 can activate the expression of epithelial–mesenchymal transition-related transcription factors such as Snail, Twist, and ZEB1, promoting the transition of cells from an epithelial phenotype to a mesenchymal phenotype [108]. Additionally, STAT3 can cooperate with other key transcription factors to collectively regulate cell phenotypic switching. Studies have shown that STAT3 and the transcription factor C/EBPβ can serve as co-promoters and master regulators to jointly drive the mesenchymal transition process [109].

The regulation of metabolic reprogramming by STAT3 is an important component of its canonical functions. In VSMCs, PDGF-BB stimulation can induce aerobic glycolysis, also known as the Warburg effect, accompanied by increased glucose uptake and upregulated expression of hexokinase 2 (HK2). STAT3 activation is a key event in PDGF-BB-induced HK2 upregulation and enhanced glycolytic activity, whereas I3MO can suppress glycolytic flux by blocking the STAT3/HK2 signaling axis [110]. In pulmonary artery smooth muscle cells under hypoxic conditions, JMJD1C can induce the expression of glycolytic enzymes such as HK2, PGK1, and LDHA, as well as lactate accumulation, by activating STAT3 signaling, and STAT3 overexpression can reverse the inhibition of glycolysis caused by JMJD1C knockdown [111]. Meanwhile, researchers have confirmed in smooth muscle cell-specific PKM2 knockout mice that nuclear-localized PKM2 can physically interact with STAT3 to cooperatively regulate the transcription of GLUT1 and LDHA, revealing a direct linkage of the PKM2/STAT3 transcriptional regulatory axis in glycolytic metabolism [112]. Additionally, Albasanz-Puig et al. found in human coronary artery smooth muscle cells that STAT3 can also indirectly promote VEGF transcription by positively regulating HIF-1α expression [113]. These lines of evidence collectively demonstrate that STAT3 plays a critical role in the metabolic reprogramming process of cells adapting to hypoxic or nutrient-stressed microenvironments through two metabolic regulatory pathways: HK2/PKM2/glycolysis and HIF-1αVEGF.

#### 2.3.2. Non-Canonical Functions of STAT3

The non-canonical functions of STAT3 refer to its activities independent of its nuclear transcriptional function, typically representing the direct actions of STAT3 within mitochondria, the endoplasmic reticulum, and the cytoplasm. These functions do not rely on its classical transcriptional regulatory activity but are instead mediated through subcellular localization and protein–protein interactions [114,115,116].

The non-canonical actions of STAT3 are fundamentally defined by its specific subcellular compartments [117]. Spatially restricted organelle localization dictates the direct molecular interactors and functional outputs of extranuclear STAT3 [118]. Mitochondrial STAT3 (mitoSTAT3) represents one of the most widely attended non-canonical functions, and its execution is precisely achieved through subcellular localization. Upon phosphorylation at the Ser727 site, STAT3 can translocate into the interior of mitochondria, where it is termed mitoSTAT3 [119,120,121]. Within mitochondria, mitoSTAT3 no longer functions as a transcription factor to regulate gene expression but directly participates in regulating core mitochondrial functions. Research indicates that mitoSTAT3 can enhance the activity of mitochondrial complex I, thereby optimizing the electron transport chain (ETC) activity and ATP synthesis efficiency of the cell. By modulating the ETC, mitoSTAT3 helps reduce the excessive generation of reactive oxygen species and participates in regulating Ca 2+ homeostasis, which plays an important role in maintaining normal mitochondrial function [116,122]. Endoplasmic Reticulum STAT3 (ERSTAT3) refers to STAT3 that localizes to the endoplasmic reticulum membrane and functions within the endoplasmic reticulum [123,124]. In CVDs, it regulates inter-organelle calcium signaling through its transcription-independent non-canonical function, which is of great significance for maintaining cardiomyocyte survival. Its core protective efficacy lies in preventing mitochondrial calcium overload, thereby inhibiting cardiomyocyte apoptosis. Specifically, ERSTAT3 interacts with IP3R3 to promote its proteasomal degradation, thereby preventing excessive calcium flux from the ER to mitochondria and suppressing apoptosis [125,126].

Protein–protein interactions refer to the process where two or more protein molecules bind together in three-dimensional space through non-covalent bonds such as hydrogen bonds, ionic bonds, and van der Waals forces to form complexes and jointly execute specific functions [127]. The mitochondrial complex I subunit gene associated with retinoid-IFN-induced mortality-19 (GRIM-19) is a key component of mitochondrial complex I, which can directly bind to STAT3 and act as a molecular chaperone to recruit STAT3 into mitochondria. This process is accomplished through the interaction between GRIM-19 and the mitochondrial outer membrane translocase Tom20, which imports STAT3 into the mitochondrial interior [128]. Once STAT3 enters mitochondria, it transitions into mitoSTAT3, thereby executing roles such as participating in the regulation of Ca2+ homeostasis [116]. In addition to GRIM-19, STAT3 can directly interact with Stathmin to regulate cellular microtubule stability [129]. Stathmin is the principal regulatory protein of microtubule dynamics, and STAT3 inhibits its microtubule-destabilizing activity through direct binding, thereby maintaining the integrity of the microtubule network [130]. Furthermore, STAT3 can interact with double-stranded RNA-dependent protein kinase R (PKR) to participate in autophagy regulation. PKR is a key regulator of the autophagic process, and STAT3 directly binds to PKR to inhibit its kinase activity, thereby blocking the overactivation of autophagy and exerting cytoprotective effects [131,132].

## 3. Regulation of VSMCs Biological Functions by the STAT3 Signaling Pathway in CVDs

In the initiation and progression of CVDs, the regulation of VSMCs biological functions by STAT3 signaling plays a critical role, mainly encompassing the modulation of VSMCs phenotypic switching, proliferation and migration, apoptosis and pyroptosis, ECM remodeling, and metabolic reprogramming [50].

### 3.1. The Role of STAT3 in VSMCs Phenotypic Switching

As the predominant cell type within the vascular wall, VSMCs exhibit remarkable phenotypic plasticity, capable of switching between the contractile and synthetic phenotypes, a process termed phenotypic switching [133]. Under physiological states, the majority of VSMCs exist in a contractile phenotype. Contractile VSMCs typically exhibit an elongated spindle shape, abundant cytoplasm, and contain a large number of myofilaments and dense bodies, with relatively few mitochondria and endoplasmic reticulum [134]. Under this phenotype, VSMCs highly express a variety of contractile-related proteins, mainly including α-smooth muscle actin (α-SMA), smooth muscle 22α (SM22α), smooth muscle myosin heavy chain (SM-MHC), and calponin, which are indispensable for maintaining cellular contractile function [135]. The synthetic phenotype is considered a dedifferentiated pathological phenotype, which primarily emerges under stimuli such as vascular injury, inflammation, or oxidative stress [136]. VSMCs under this phenotype are mostly oval or polygonal, with enlarged cell bodies, well-developed rough endoplasmic reticulum and Golgi apparatus, and a marked downregulation of contractile protein expression accompanied by a significantly enhanced capacity to secrete ECM components such as matrix metalloproteinases (MMPs), collagens (types I and III), and fibronectin [137].

Numerous studies have pointed out that STAT3, as a key component of the JAK2/STAT3 pathway, plays a central role in VSMCs phenotypic switching [138,139,140]. Specifically, when cytokines such as IL-6 bind to cell surface receptors, JAK2 is activated and phosphorylates STAT3 at the Tyr705 site [39,141]. Phosphorylated STAT3 subsequently homodimerizes and translocates into the nucleus, where it binds to gamma-interferon-activated sequence (GAS) elements within the promoter regions of target genes, thereby driving downstream gene transcription [142,143,144], which downregulates contractile markers and upregulates synthetic phenotypic markers in VSMCs, ultimately promoting VSMCs phenotypic switching [145].

In addition, activated STAT3 can directly suppress the expression of VSMCs contractile phenotypic markers by competing with serum response factor (SRF) or myocardin for binding to CArG elements, thereby promoting VSMCs phenotypic switching [146]. Myocardin maintains the contractile state by binding SRF to drive CArG-dependent gene transcription [147,148,149]. Activated STAT3 competitively binds CArG elements to displace the myocardin/SRF complex, thereby downregulating contractile marker expression [150].

**Figure 4:**
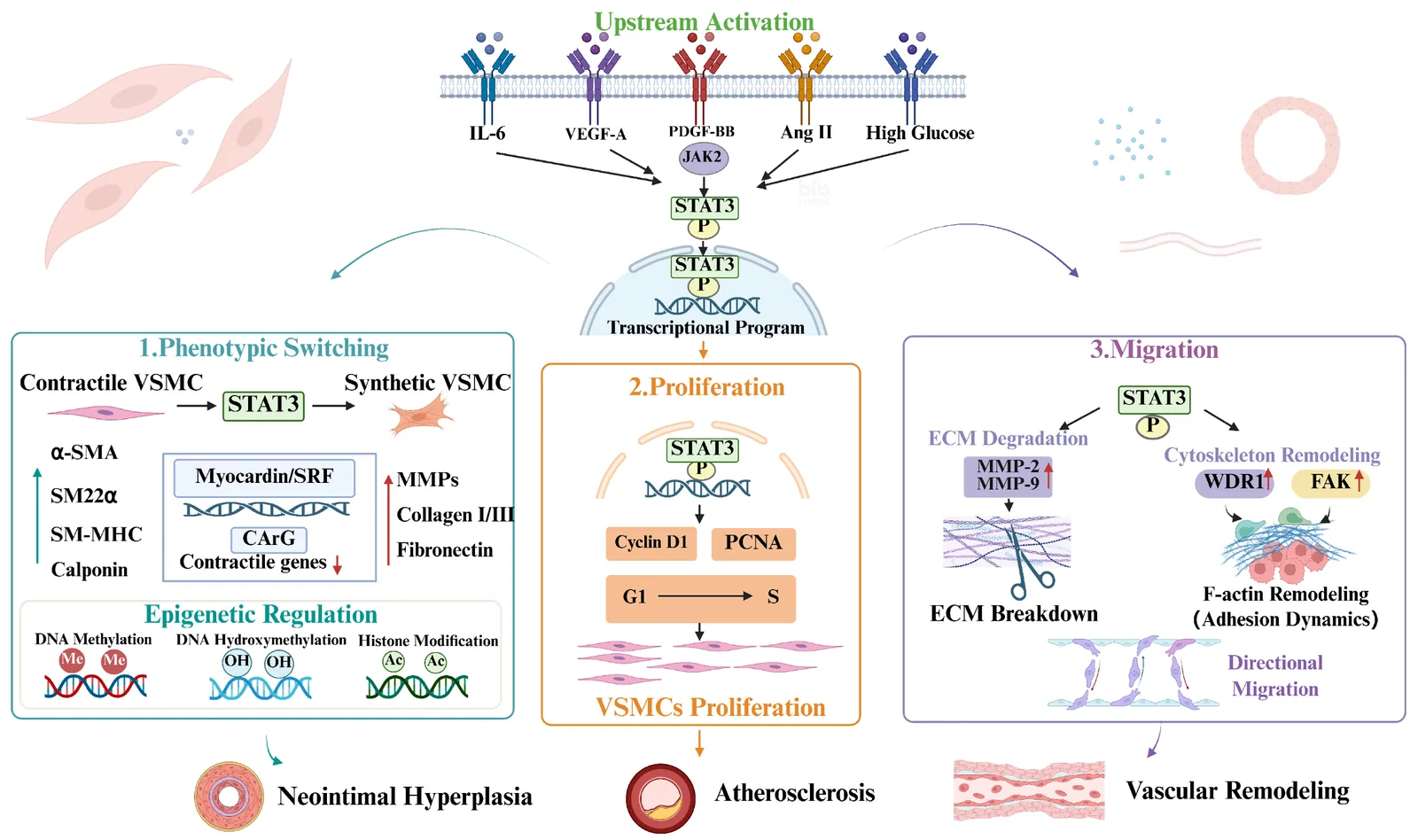
STAT3 regulates VSMCs phenotypic switching, proliferation, and migration. Multiple pathological stimuli, including IL-6, VEGF-A, PDGF-BB, Ang II, and high glucose, activate the JAK2/STAT3 signaling pathway. Activated STAT3 promotes the transition of VSMCs from a contractile to a synthetic phenotype through suppression of contractile gene expression and epigenetic remodeling. In addition, STAT3 stimulates VSMCs proliferation by inducing Cyclin D1 and PCNA expression and accelerating G1/S cell-cycle progression. STAT3 also facilitates VSMCs migration through MMP-2/9-mediated ECM degradation and WDR1- and FAK-dependent cytoskeletal remodeling. These coordinated effects contribute to neointimal hyperplasia, atherosclerosis, and vascular remodeling. The figure was created in BioRender.

STAT3 can also regulate VSMCs phenotypic switching through complex epigenetic modification mechanisms, thereby participating in the initiation and progression of various CVDs [123,148,150]. Epigenetic modification refers to the regulation of gene expression through mechanisms such as DNA methylation, histone modification, and non-coding RNAs without altering the DNA sequence, which determines cell identity and function [123,148]. DNA methylation refers to the addition of methyl groups to cytosine bases in DNA, which is typically associated with gene silencing. Research has confirmed that STAT3 can recruit DNA methyltransferases to promote DNA hypermethylation in the promoter regions of contractile genes, thereby suppressing their expression [151]. DNA hydroxymethylation (5-hydroxymethylcytosine, 5hmC) is a dynamic epigenetic modification catalyzed by Ten-Eleven Translocation (TET) family dioxygenases; serving as a critical intermediate in the active DNA demethylation pathway, its enrichment is usually tightly coupled with transcriptional activation of genes [152,153]. In vascular biology, TET2 has been established as a core epigenetic regulator determining the differentiation fate of VSMCs, mainly maintaining the smooth muscle-specific contractile phenotype by reshaping the chromatin accessibility (chromatin remodeling) of target gene promoter regions [154]. However, in pathological microenvironments such as vascular remodeling, the aberrantly activated STAT3 signaling axis may serve as a critical upstream molecular hub, leading to the loss of local genomic DNA hydroxymethylation levels by modulating the expression abundance or catalytic activity of TET enzymes, thereby epigenetically silencing contractile marker gene expression and inducing VSMCs phenotypic switching [155]. STAT3 also modulates phenotypic switching via chromatin-remodeling histone modifications [156]. STAT3 recruits HATs to enrich H3K27ac marks [157,158], thereby altering chromatin accessibility at smooth muscle-specific loci [158,159] (Figure 4).

### 3.2. The Role of STAT3 in VSMCs Proliferation

Aberrant proliferation of VSMCs is a central pathological event in CVDs such as neointimal hyperplasia after vascular injury, atherosclerosis, and hypertensive vascular remodeling [159]. Under physiological states, most VSMCs in the vascular wall remain in a quiescent phase, exhibiting extremely low proliferative activity to ensure normal vascular structure and vasomotor function; whereas under pathological stimuli, VSMCs are abnormally activated and undergo excessive proliferation, ultimately resulting in vascular wall thickening and luminal stenosis. The transcription factor STAT3 plays a core driving role in this process, directly activating the transcription of cell cycle-related target genes by integrating various extracellular pro-proliferative signals, pushing VSMCs from a quiescent state into an active proliferative cycle.

The molecular mechanisms by which STAT3 regulates VSMCs proliferation primarily involve two levels: the activation of STAT3 by multiple upstream pro-proliferative signals, and the downstream transcriptional regulation of target genes after STAT3 enters the nucleus. At the upstream level, various pro-proliferative factors activate the STAT3 signaling pathway through different molecular routes. PDGF-BB binds to its receptor PDGFR-β to activate JAK2 kinase, which in turn phosphorylates STAT3 at the Tyr705 site, forming activated STAT3 homodimers that translocate into the cell nucleus, thereby driving VSMCs proliferation [21]. Vascular endothelial growth factor A (VEGF-A) exerts pro-proliferative effects through another signaling cascade involving VEGFR2 and STAT3. Following the binding of VEGF-A to its receptor VEGFR2, the activated VEGFR2 transmits signals through a complex cascade, which subsequently activates STAT3 and promotes the upregulation of VSMCs proliferation markers such as Cyclin D1 and PCNA [160]. Under pathological states such as hypertension, AngII stimulation can activate the JAK2/STAT3 pathway, thereby enhancing NADPH oxidase activity, promoting the generation of superoxide anions and hydrogen peroxide, and inducing a significant elevation in nitrative and oxidative stress levels. The elevated oxidative stress further upregulates the expression of Giα protein and various cyclins such as Cyclin D1 and Cyclin B1, ultimately leading to the excessive proliferation of VSMCs [161]. At the downstream level, activated STAT3 drives VSMCs proliferation by transcriptionally regulating the expression of cell cycle-related genes. Upon entering the nucleus, STAT3 directly binds to the promoter region of the Cyclin D1 gene to activate its transcription [90,162]. As a key regulator of the G1 phase cell cycle, Cyclin D1 plays a core role in driving the cell cycle transition from G1 to S phase, and its upregulation is a crucial downstream event in STAT3-mediated VSMCs proliferation [163,164]. Targeting STAT3 with antisense oligonucleotides can significantly inhibit VSMCs growth and downregulate the expression of p-STAT3 and Cyclin D1, further confirming that Cyclin D1 is a key downstream target gene in STAT3-mediated VSMCs proliferation [165].

### 3.3. The Role of STAT3 in VSMCs Migration

The directional migration of VSMCs from the tunica media across the internal elastic lamina into the intima is a common core cellular event in neointima formation after vascular injury, atherosclerotic plaque progression, and restenosis after percutaneous coronary intervention [166,167]. The migration process involves three sequential steps: phenotypic switching of cells from the contractile to synthetic phenotype, enzymatic degradation of the ECM barrier, and directional displacement driven by cytoskeletal reorganization [168,169,170]. STAT3 is considered the signaling hub for VSMCs migration, playing a critical regulatory role in directing this process.

Under various pathological stimuli, upstream signals activate STAT3 phosphorylation to initiate the VSMCs migration program. PDGF-BB is the most classical pro-migratory factor for VSMCs. Studies have found that inhibiting JAK2 and STAT3 using dominant-negative mutants can almost completely block PDGF-BB-induced VSMCs motility, directly confirming the necessity of PDGF-BB and the JAK2/STAT3 signaling axis in VSMCs migration [171]. Beyond PDGF-BB, Ang II can similarly activate the STAT3 signaling pathway by binding to AT1 receptors on the surface of VSMCs, thereby facilitating VSMCs migration and the development of vascular lesions [172,173]. Furthermore, a high-glucose environment can also induce VSMCs migration by activating the Src/STAT3 pathway [174]. Activated STAT3 primarily drives the physical displacement of VSMCs through the classical transcription-dependent pathway. Upon entering the nucleus to exert its function as a transcription factor, STAT3 directly upregulates the transcription of matrix metalloproteinase-2 (MMP-2) and matrix metalloproteinase-9 (MMP-9), which is the molecular prerequisite for VSMCs to cross the basement membrane and internal elastic lamina. In VSMCs, siRNA knockdown of STAT3 or blockade of upstream receptors significantly inhibits the transcriptional upregulation of MMP-2/9 and the subsequent migratory capacity, indicating that STAT3 degrades the ECM barrier by modulating the transcriptional levels of MMP-2/9, thereby clearing physical obstacles for the directional displacement of VSMCs [175]. In rat aortic VSMCs, cigarette smoke extract can induce the secretion of MMP-2 and MMP-9 through the JAK2/STAT3 pathway, and transfection with JAK2 or STAT3 siRNA can significantly reduce MMP-2/9 production, further supporting the critical position of STAT3 in MMP transcription and VSMCs migration regulation [176]. Directional migration requires coordinated cytoskeletal reorganization driven by lamellipodial F-actin dynamics and focal adhesion turnover [168,177,178]. STAT3 directly upregulates WDR1 transcription, facilitating cofilin-mediated actin turnover to promote VSMC motility and neointimal formation [179]. In addition, focal adhesion kinase (FAK), as a core downstream effector molecule of integrin signaling, is functionally coupled with STAT3 signaling. Studies have shown that FAK phosphorylation can significantly affect VSMCs migration by influencing actin cytoskeletal remodeling. In that study, the natural product Rubiarbonone C significantly reduced PDGF-BB-induced F-actin reorganization by inhibiting the phosphorylation of FAK, MAPK, and STAT3 Tyr705, and ultimately suppressed VSMCs migration, indicating that FAK and STAT3 constitute a signaling node regulating actin cytoskeletal reorganization [180].

### 3.4. The Role of STAT3 in VSMCs Apoptosis and Pyroptosis

The STAT3 signaling pathway plays a critical role in regulating the apoptosis and pyroptosis of VSMCs, two distinct modes of cell death that govern the progression of pathological remodeling and inflammatory lesions following vascular injury. Specifically, STAT3 predominantly suppresses VSMCs apoptosis through the transcriptional activation of anti-apoptotic proteins, thereby promoting neointimal hyperplasia [181,182]. Conversely, STAT3-mediated activation of the NOD-like receptor thermal protein domain associated protein 3 (NLRP3) inflammasome and the GSDMD/GSDME-dependent pyroptotic pathways exacerbate vascular inflammation and lesion progression [183,184].

As a key transcription factor regulating VSMCs apoptosis, STAT3 can directly bind to the promoter regions of anti-apoptotic genes such as Bcl-2, Bcl-xL, Mcl-1, and Survivin to transcriptionally activate their expression, thereby blocking the transmission of apoptotic signals at multiple levels [143,185]. During neointima formation following vascular injury, transient activation of STAT3 upregulates Mcl-1 by 8-fold and Bcl-xL by 5-fold, whereas the pro-apoptotic protein Bax is only slightly elevated [186]. To further elucidate the central role of STAT3 in modulating VSMCs survival, researchers utilized an adenoviral vector to construct a dominant-negative STAT3 (AxCAdnSTAT3). Dominant-negative STAT3 is a mutant in which tyrosine 705 (Tyr705) of STAT3 is substituted with phenylalanine (Y705F); although this mutant can normally bind to phosphorylated receptors and be recruited to signaling complexes, it cannot be activated due to the lack of a phosphorylation site, and it forms heterodimers with endogenous STAT3 that lack DNA-binding capacity, thereby competitively inhibiting the dimerization, nuclear translocation, and target gene transcription of endogenous STAT3 [187]. Overexpression of AxCAdnSTAT3 in balloon-injured rat carotid arteries not only completely blocked STAT3 phosphorylation but also significantly increased the apoptotic index of neointimal VSMCs and markedly reduced the intima-to-media area ratio [186]. This indicates that the blockade of STAT3 signaling significantly promotes VSMCs apoptosis and effectively suppresses excessive neointimal hyperplasia, directly confirming the core driving role of STAT3 in anti-apoptotic regulation in VSMCs. In atherosclerosis, single-cell RNA sequencing analysis revealed that IL-1β promotes the adhesion, inflammatory response, and apoptosis of macrophage-like VSMCs in a STAT3-dependent manner, thereby influencing the plaque microenvironment and disease progression [188]. These studies demonstrate that STAT3-mediated anti-apoptotic regulation in VSMCs is a critical molecular mechanism underlying pathological vascular remodeling and atherosclerotic progression.

**Figure 5:**
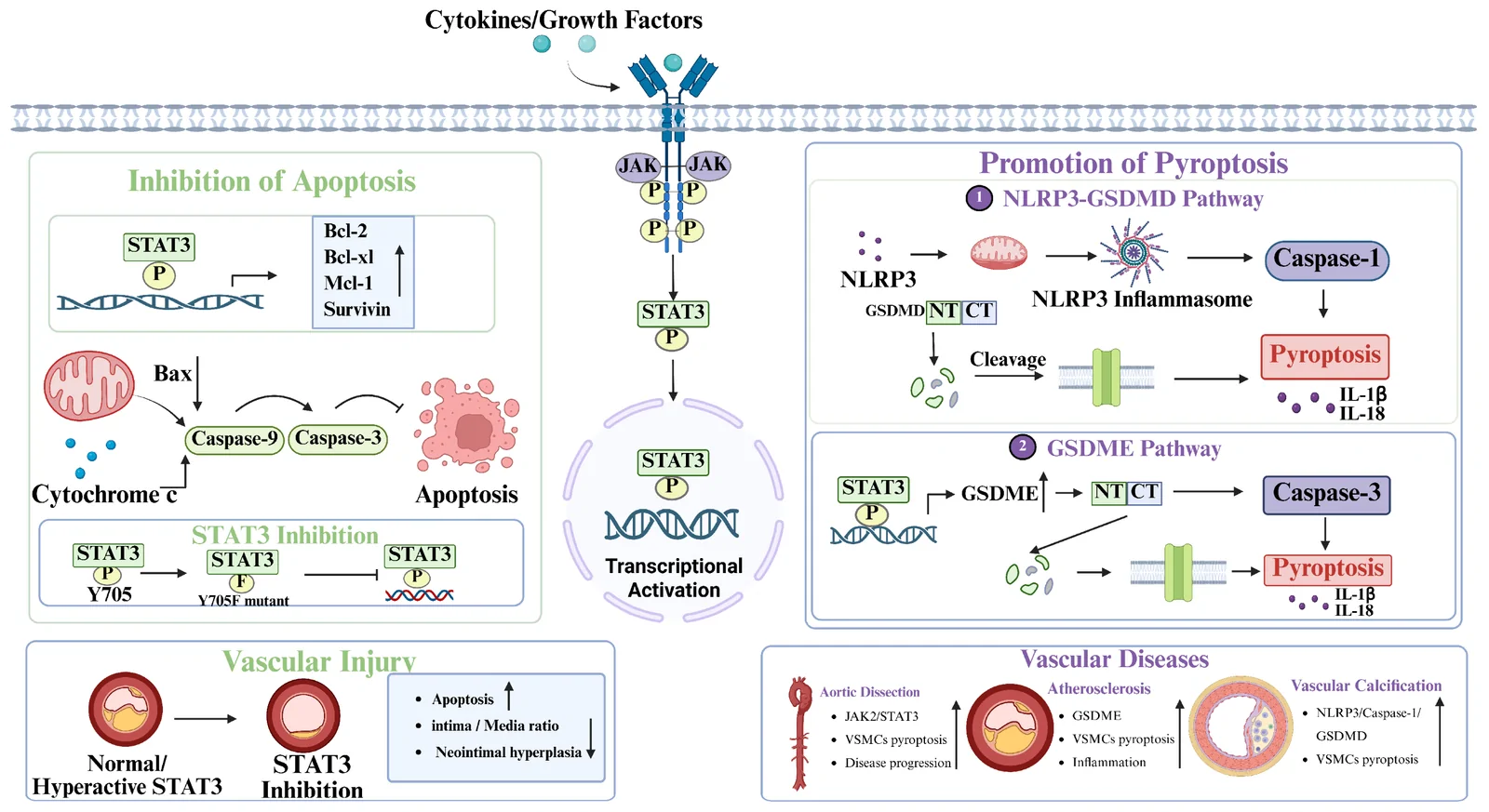
Molecular mechanisms by which STAT3 regulates apoptosis and pyroptosis in VSMCs. STAT3 plays a dual role in regulating the fate of VSMCs, contributing to vascular remodeling and inflammatory vascular diseases by suppressing apoptosis while promoting pyroptosis. Upon stimulation by cytokines or growth factors, activation of the JAK/STAT3 signaling pathway induces STAT3 phosphorylation, followed by its nuclear translocation and transcriptional activation of downstream target genes. In the anti-apoptotic pathway, STAT3 directly promotes the transcription of anti-apoptotic genes, including Bcl-2, Bcl-xL, Mcl-1, and Survivin, thereby suppressing mitochondrial apoptotic signaling, reducing cytochrome c release and subsequent Caspase-9/Caspase-3 activation, and ultimately enhancing VSMCs survival and neointimal formation following vascular injury. In contrast, the dominant-negative STAT3 mutant (Y705F) inhibits STAT3-dependent transcriptional activity, leading to increased VSMCs apoptosis and attenuation of pathological neointimal hyperplasia. In the pyroptotic pathway, STAT3 promotes inflammatory cell death through two major mechanisms. First, STAT3 interacts with NLRP3 and facilitates NLRP3 inflammasome assembly and activation, resulting in Caspase-1 activation and subsequent cleavage of gasdermin D (GSDMD). The released pore-forming GSDMD N-terminal fragment induces membrane permeabilization and promotes the secretion of pro-inflammatory cytokines, including IL-1β and IL-18. Second, STAT3 directly enhances the transcription of gasdermin E (GSDME), which is subsequently cleaved by Caspase-3 to generate the pore-forming GSDME N-terminal fragment, thereby triggering pyroptotic cell death. STAT3-mediated pyroptotic signaling has been implicated in the pathogenesis of multiple vascular disorders, including AD, atherosclerosis, and VC. The figure was created in BioRender.

Pyroptosis is a programmed inflammatory cell death mode mediated by inflammasomes, characterized primarily by plasma membrane pore formation, cellular swelling and rupture, and the massive release of pro-inflammatory cytokines such as IL-1β and IL-18, serving as a core driver in maintaining and amplifying the vascular inflammatory microenvironment [189,190]. STAT3 acts as a key driving molecule in this process. Luo et al. discovered that under stimulation by various NLRP3 agonists, STAT3 directly interacts with NLRP3 and undergoes phosphorylation at the Ser727 site, subsequently mediating the co-translocation of STAT3 and its bound NLRP3 to mitochondria, thereby facilitating the assembly and activation of the NLRP3 inflammasome [183]. Upon activation, the NLRP3 inflammasome recruits and activates caspase-1, which specifically cleaves the linker region between the N-terminal pore-forming domain and the C-terminal autoinhibitory domain of the GSDMD protein, releasing the pore-forming GSDMD N-terminal domain (GSDMD-NT) [184]. This domain oligomerizes to form pores on the plasma membrane, leading to membrane rupture and the massive release of inflammatory contents, ultimately inducing pyroptosis in VSMCs [189]. Disrupting the interaction between STAT3 and NLRP3 impairs the mitochondrial localization of NLRP3 and specifically suppresses NLRP3 inflammasome activation, thereby inhibiting VSMCs pyroptosis [183]. Additionally, STAT3 can positively regulate the transcriptional expression of GSDME by directly interacting with multiple binding sites on its gene promoter [191]. GSDME is another key member of the Gasdermin family of pyroptosis executioner proteins, which is primarily cleaved and activated by caspase-3 rather than caspase-1 [192]. Activated caspase-3 specifically cleaves the junction between the N-terminal pore-forming domain and the C-terminal autoinhibitory domain of GSDME, releasing the pore-forming GSDME-NT, which forms pores on the plasma membrane to ultimately induce cellular pyroptosis [193]. In CVDs, STAT3-mediated VSMCs pyroptosis has been confirmed to participate in multiple vascular pathological processes. Studies have shown that in animal models of AD, the expression of leukocyte immunoglobulin-like receptor B4 (LILRB4) is significantly elevated; knockdown of LILRB4 can significantly reduce the level of VSMCs pyroptosis by inhibiting the JAK2/STAT3 signaling pathway, promote the phenotypic transition of VSMCs toward the contractile state, and stabilize the ECM, thereby suppressing AD progression [190]. Meanwhile, in GSDME/ApoE double-knockout mice induced by a high-fat diet, the atherosclerotic lesion area and inflammatory responses were significantly attenuated, revealing that STAT3 can positively regulate GSDME transcription to promote GSDME-mediated VSMCs pyroptosis and inflammation [194]. In VC, pyroptosis-related molecules such as NLRP3, caspase-1, and GSDMD are significantly upregulated in high-phosphate-induced calcified VSMCs; blocking caspase-1 activity can attenuate VSMCs pyroptosis and calcium deposition by suppressing STAT3 activation [195]. These lines of evidence collectively demonstrate that STAT3, by mediating NLRP3 inflammasome activation and GSDMD cleavage-driven pyroptotic signals, plays a pivotal pathological role in driving vascular lesions such as AD (Figure 5).

### 3.5. The Role of STAT3 in VSMCs ECM Synthesis

The ECM of the vascular wall is a dynamic complex that maintains vascular structural integrity and mechanical properties, and its metabolic imbalance represents a common pathological foundation for vascular remodeling diseases such as hypertension, atherosclerosis, and AD [196]. VSMCs are the primary source of ECM proteins within the vascular tunica media; under a synthetic phenotype, they abundantly produce structural ECM components including collagens (types I and III), fibronectin (FN), and elastin, and integrate them into the vascular wall [197]. The maintenance of ECM homeostasis depends on the net balance between the synthetic and degradative arms, the former being dictated by the transcriptional synthesis rate of ECM proteins, whereas the latter is governed by the activity balance of MMPs and their endogenous tissue inhibitors of metalloproteinases (TIMPs) [198]. STAT3 exerts significant regulatory effects on both the synthetic and degradative arms, and the net effect between its pro-synthetic and pro-degradative actions collectively constitutes the molecular hub determining the ultimate fate of the vascular wall ECM.

In the ECM synthetic arm, STAT3 drives transcription by binding to the regulatory regions of ECM genes, serving as a core transcriptional mechanism that governs VSMCs ECM synthesis. STAT3 can directly bind to the distal enhancer of the collagen type I α chain gene (COL1A2) and cooperate with JunB to facilitate the recruitment of RNA polymerase II to activate its transcription, while also influencing collagen production at the post-transcriptional level by modulating collagen mRNA stability [199]. In vivo, a smooth muscle-specific SOCS3 knockout mouse model (smSOCS3-KO) provides a critical validation of the causal relationship between STAT3 and ECM deposition, demonstrating that SOCS3 deficiency enhances STAT3 activation in VSMCs, increases collagen fiber deposition in the aortic adventitia, significantly elevates the tensile strength of the aortic wall, and attenuates the development of AD induced by β-aminopropionitrile combined with Ang II [200]. Furthermore, in human AD surgical specimens, STAT3 phosphorylation signals are robustly detected at the dissection site, whereas the STAT3 phosphorylation level is markedly diminished in regions where collagen deposition has been completed, suggesting that temporal changes in STAT3 activity are closely linked to the ECM stabilization phase following the completion of collagen deposition [200]. The synergistic action between STAT3 Lys685 acetylation and Tyr705 phosphorylation serves as the core molecular switch determining its pro-ECM synthetic transcriptional activity, which is bidirectionally regulated by WWP2 and Septin4. Specifically, WWP2 (a HECT domain-containing E3 ubiquitin ligase) interacts with SIRT1 and STAT3 to form a ternary complex, inhibiting SIRT1-mediated deacetylation of STAT3, thereby increasing the levels of STAT3 Lys685 acetylation and Tyr705 phosphorylation and reinforcing STAT3 transcriptional activity [201]. Hyperhomocysteinemia (Hcy) can also activate the SIRT1/STAT3 pathway by upregulating WWP2 expression, subsequently promoting ECM remodeling in VSMCs [22]. Conversely, Septin4 acts as a negative regulator of this pathway by forming a ternary complex with SIRT1 and STAT3 to enhance the interaction between SIRT1 and STAT3, promoting SIRT1-mediated STAT3 Lys685 deacetylation and Tyr705 dephosphorylation, thereby reducing STAT3 transcriptional activity [202]. WWP2 and Septin4 modulate STAT3 acetylation and phosphorylation levels in diametrically opposite manners to directly control the transcriptional expression intensity of STAT3 toward ECM genes, meaning that when WWP2 activity predominates, STAT3 acetylation and phosphorylation levels elevate, enhancing ECM gene transcription, whereas when Septin4 activity predominates, SIRT1-mediated deacetylation and dephosphorylation are accelerated, dampening ECM gene transcription.

**Figure 6:**
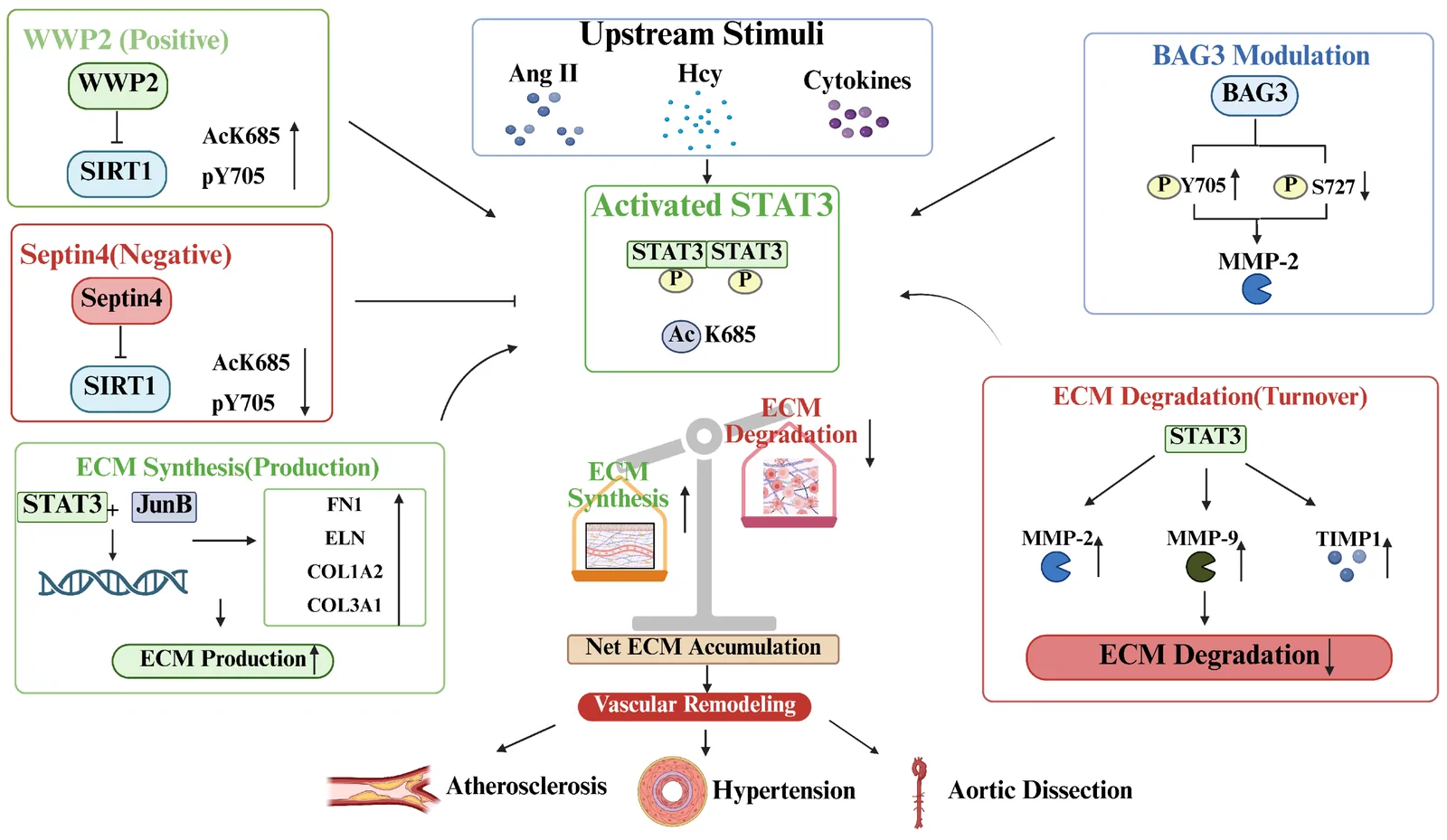
Mechanisms by which STAT3 regulates ECM homeostasis in VSMCs. Various upstream stimuli, including AngII, homocysteine (Hcy), and inflammatory cytokines, activate STAT3 signaling in VSMCs. The transcriptional activity of STAT3 is cooperatively regulated by Lys685 acetylation (AcK685) and Tyr705 phosphorylation (pY705). WWP2 enhances STAT3 activation by suppressing SIRT1-mediated deacetylation, whereas Septin4 exerts an opposing effect by facilitating the interaction between SIRT1 and STAT3. On the ECM synthesis side, activated STAT3 cooperates with JunB to promote the transcription of ECM-related genes, including COL1A2, COL3A1, FN1, and ELN, thereby enhancing ECM production. On the ECM degradation side, STAT3 regulates the expression of MMP-2 and MMP-9 and their endogenous inhibitor TIMP1. Because the stimulatory effect on TIMP1 predominates, the overall consequence is reduced ECM degradation. In addition, BAG3 modulates ECM turnover by differentially regulating STAT3 Tyr705 and Ser727 phosphorylation and subsequently affecting MMP-2 expression. Through coordinated regulation of ECM synthesis and degradation, STAT3 promotes net ECM accumulation, drives vascular remodeling, and contributes to the progression of vascular diseases, including atherosclerosis, hypertension, and AD. The figure was created in BioRender.

In the ECM degradative arm, the activity balance between MMPs and TIMPs directly determines the net degradation rate of the ECM, and STAT3 finely tunes the direction of this balance through differential transcriptional regulation of both components. In vascular wall ECM metabolism, MMPs are responsible for degrading ECM protein components, whereas TIMPs inhibit their proteolytic activity by forming 1:1 complexes with MMPs, and their relative activities dictate whether the ECM tends toward degradation or accumulation [203]. The regulation of MMPs and TIMPs by STAT3 does not simply promote degradation in a unidirectional manner but manifests as a fine-tuned balanced regulation. STAT3 can simultaneously enhance the promoter activities of both the MMP-9 and TIMP1 genes to promote their transcription and expression; notably, its inductive effect on TIMP1 is markedly stronger than that on MMP-9, culminating in a net effect that favors attenuated ECM degradation capacity and the accumulation of ECM components [204,205]. Additionally, BAG3 influences the ECM balance by differentially regulating the phosphorylation levels of the STAT3 Tyr705 and Ser727 sites; specifically, BAG3 increases the level of p-STAT3 Tyr705 while simultaneously decreasing the level of p-STAT3 Ser727, thereby modulating MMP-2 expression and promoting VSMCs migration and aberrant ECM metabolism during diabetic vascular remodeling [206]. These findings unveil a key characteristic of STAT3 in regulating ECM degradation, wherein its upregulation of TIMP1 outweighs that of MMP-9, biasing the net effect of the degradative arm toward the inhibition of ECM degradation, thereby synergizing with the pro-transcriptional function of the synthetic arm to collectively drive net ECM deposition (Figure 6).

### 3.6. The Role of STAT3 in VSMCs Metabolic Reprogramming

The phenotypic plasticity of VSMCs is a core progression in various CVDs, such as atherosclerosis and vascular remodeling, with metabolic reprogramming serving as one of the driving factors behind this transition [207]. STAT3 regulates VSMCs metabolism through multiple mechanisms, including direct and indirect influences on glycolysis, mitochondrial function, and synergistic interactions with other signaling pathways. As a transcription factor, upon nuclear translocation, STAT3 can bind to the promoter regions of various key glycolytic enzyme genes, thereby upregulating their expression and promoting aerobic glycolysis, also known as the Warburg effect, in VSMCs. In atherosclerosis, VSMCs undergo a metabolic shift resembling the Warburg effect, supporting their proliferation, migration, and phenotypic modulation by enhancing glycolytic flux [208]. This metabolic reprogramming provides the necessary energy and biosynthetic precursors for the synthetic phenotype of VSMCs [209]. Studies have revealed that STAT3 can upregulate the expression of glycolytic enzymes such as HK2, pyruvate kinase M2 (PKM2), and lactate dehydrogenase A (LDHA), which are key rate-limiting enzymes in the glycolytic pathway. HK2 catalyzes the first step of glycolysis, phosphorylating glucose into glucose-6-phosphate. PKM2 represents a critical regulatory node in the glycolytic pathway, and its M2 isoform is highly expressed in rapidly proliferating cells, promoting the accumulation of glycolytic intermediates to provide substrates for biosynthesis [210]. LDHA is responsible for converting pyruvate into lactate while regenerating NAD+, thereby sustaining high-flux glycolysis. Krüppel-like factor 4(KLF4) plays a key role in VSMCs phenotypic switching via PFKFB3-driven glycolysis, and the activation of STAT3 may synergize with this process [211].

STAT3 not only impacts glycolysis but also participates in the regulation of mitochondrial function. Mitochondria serve as the energy powerhouses of the cell, and their function and dynamics are of paramount importance in VSMCs phenotypic switching and CVDs [212]. Research indicates that STAT3 can interact with ETC complexes I and II on the inner mitochondrial membrane, thereby influencing their activities. Persistent activation of STAT3 may suppress the excessive production of reactive oxygen species (ROS) and contribute to maintaining the mitochondrial membrane potential [213]. However, chronic or aberrant activation of STAT3 may also induce mitophagy and mitochondrial DNA damage, resulting in declined oxidative phosphorylation efficiency. Mitochondrial dysfunction accelerates the osteogenic differentiation of VSMCs in chronic kidney disease-associated VC [214].

**Figure 7:**
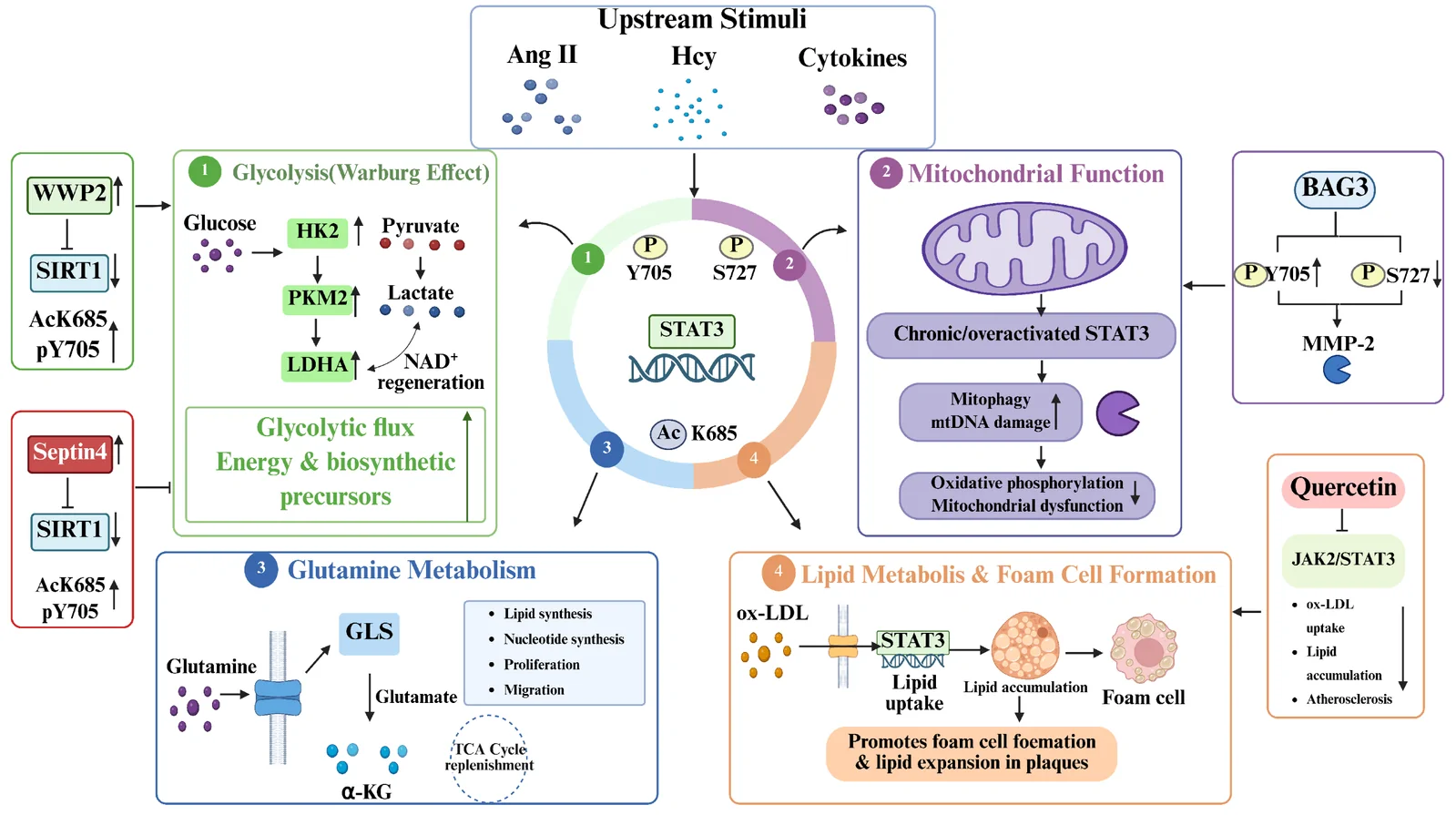
STAT3-mediated metabolic reprogramming in VSMCs. STAT3 functions as a central regulator of metabolic reprogramming in VSMCs by integrating multiple upstream stimuli, including Ang II, Hcy, and inflammatory cytokines. Activated STAT3 promotes a Warburg-like glycolytic phenotype through upregulation of key glycolytic enzymes, including HK2, PKM2, and LDHA, thereby enhancing glycolytic flux and supporting the energy demands of VSMCs phenotypic switching, proliferation, and migration. STAT3 also regulates mitochondrial homeostasis and glutamine metabolism by modulating mitochondrial function and GLS-dependent α-KG production, which replenishes the TCA cycle and supports biosynthetic processes. In addition, STAT3 promotes ox-LDL uptake, lipid accumulation, and foam cell formation, contributing to atherosclerotic plaque progression. The transcriptional activity of STAT3 is further modulated by WWP2, Septin4, BAG3, and pharmacological inhibitors such as quercetin. Collectively, STAT3 orchestrates multiple metabolic pathways that drive VSMCs dysfunction and vascular disease progression. The figure was created in BioRender.

Concurrently, STAT3 can also modulate glutamine metabolism. Glutamine serves as an essential substrate for cell proliferation and biosynthesis. STAT3 can promote the expression of glutaminase (GLS) and enhance the conversion of glutamine into α-ketoglutarate (α-KG), thereby replenishing tricarboxylic acid (TCA) cycle intermediates to support lipid and nucleotide synthesis. The acceleration of glutaminolysis is intimately associated with VSMCs proliferation and migration, and inhibiting glutaminolysis can simultaneously attenuate both glycolysis and oxidative phosphorylation, thereby suppressing VSMCs proliferation and migration [215]. Furthermore, STAT3 participates in regulating lipid metabolism and foam cell formation in VSMCs. Within atherosclerotic plaques, VSMCs can engulf oxidized low-density lipoprotein (ox-LDL) and transdifferentiate into macrophage-like foam cells, a process that acts as a core driver of plaque progression and lipid expansion. PIAS3 is a negative regulator of the JAK/STAT3 signaling pathway, and its expression level is significantly reduced in atherosclerotic lesions. Studies have confirmed that PIAS3 overexpression effectively suppresses ox-LDL-induced inflammatory responses, lipid accumulation, and cell proliferation in VSMCs, indicating that the overactivation of STAT3 signaling facilitates aberrant lipid deposition in VSMCs, a process inhibited by PIAS3 via its negative regulation of STAT3 activity [216]. In an ApoE−/− mouse model of atherosclerosis, the deficiency in PIAS3 markedly exacerbates plaque formation and lipid accumulation [217]. Conversely, quercetin can block KLF4-mediated phenotypic transition of VSMCs into macrophage-like cells by inhibiting the JAK2/STAT3 pathway, reducing intracellular DiI-ox-LDL uptake and lipid accumulation, thereby effectively preventing the formation of VSMC-derived foam cells; in high-fat diet-fed ApoE−/− mice, quercetin significantly attenuates atherosclerotic lesion area and lipid deposition [218] (Figure 7).

## 4. Signaling Pathways Involved in STAT3 Regulation of VSMCs Biological Functions

The regulation of VSMCs biological functions is mediated through the activation of multiple interconnected cellular signaling pathways. As a point of convergence for several signaling cascades, STAT3 plays an exceptionally critical role in the biological regulation of VSMCs. The signaling networks involving STAT3 primarily encompass the JAK2/STAT3 signaling pathway, the Src/STAT3 signaling pathway, and the SIRT1/STAT3 signaling pathway, among others.

### 4.1. The JAK2/STAT3 Signaling Pathway

The JAK2/STAT3 signaling pathway serves as a core signaling axis regulating VSMCs proliferation, anti-apoptosis, migration, and phenotypic switching, playing a pivotal role in vascular remodeling and the pathogenesis of various vascular diseases [219]. In VSMCs, the activation of this pathway is triggered by the binding of diverse vasoactive factors and growth factors—including Ang II, ox-LDL, and PDGF-BB—to their respective cell-surface receptors. This engagement leads to JAK2 autophosphorylation, phosphorylation at the STAT3 Tyr705 site, and subsequent STAT3 dimerization and nuclear translocation, ultimately binding to the GASs within target gene promoter regions to initiate downstream transcriptional programs [220].

At the level of phenotypic modulation, the activation of the JAK2/STAT3 pathway suppresses the expression of contractile markers (such as α-SMA, SM22α, and calponin) while upregulating synthetic markers (such as OPN and Ki-67), thereby driving the transition of VSMCs from a contractile to a synthetic state [221]. Regarding the regulation of proliferation and migration, Ang II can induce the overexpression of the Giα protein via the JAK2/STAT3 pathway, promoting excessive VSMCs proliferation [222]. In the context of inflammation and oxidative stress, RAGE modulates mitochondrial dynamics through the JAK2/STAT3 pathway during diabetic vascular remodeling, thereby affecting VSMCs functions [222]. High phosphate can also induce sustained activation of JAK2/STAT3 signaling, mediating inflammatory responses in VSMCs [223]. These lines of evidence demonstrate that under metabolic pathological conditions such as diabetes and hyperphosphatemia, the JAK2/STAT3 pathway facilitates pathological phenotypic switching of VSMCs by driving inflammation and oxidative stress. Furthermore, the JAK2/STAT3 signaling pathway can synergize with other important transcription factors, such as NF-κB and HIF-1α, to amplify inflammatory and hypoxic responses, thereby further impacting phenotypic remodeling in VSMCs.

### 4.2. The Src/STAT3 Signaling Pathway

The Src/STAT3 signaling pathway represents another critical signaling axis regulating VSMCs proliferation, migration, anti-apoptosis, and phenotypic switching, playing an important role in the initiation and progression of various vascular diseases, including vascular injury remodeling, atherosclerosis, hypertension, and restenosis [224]. Within VSMCs, a multitude of stimulatory factors can activate the Src/STAT3 pathway to initiate downstream transcriptional programs. Under high-glucose conditions, the protein levels of both phosphorylated Src and phosphorylated STAT3 are significantly elevated, and high-glucose-induced VSMCs proliferation and migration are primarily achieved through the activation of the Src/STAT3 signaling pathway [143]. As a potent inducer of VSMCs proliferation, PDGF-BB can likewise activate the Src/STAT3 pathway, with 12/15-lipoxygenase confirmed as a critical signaling hub for PDGF-BB-induced STAT3 activation [225]. The core mechanism of this pathway relies on Src, a non-receptor tyrosine kinase, undergoing autophosphorylation upon the activation of various receptors and directly phosphorylating the STAT3 Tyr705 site or indirectly promoting STAT3 phosphorylation via JAKs. Subsequently, phosphorylated STAT3 forms dimers and translocates into the nucleus, binding to DNA sequences within the promoter regions of target genes to regulate the transcription of genes associated with proliferation, survival, inflammation, and phenotypic switching, such as Bcl-xL, Cyclin D1, MMP-2, and SOCS3, thereby directly governing diverse biological behaviors of VSMCs [20,226].

### 4.3. The SIRT1/STAT3 Pathway

The SIRT1/STAT3 pathway serves as a critical regulatory node connecting deacetylation modifications with STAT3 signal transduction. As a NAD+-dependent deacetylase, SIRT1 can directly deacetylate STAT3, suppressing its transcriptional activation capacity and thereby modulating pathological processes such as inflammation and proliferation in VSMCs. In the hypoxic microenvironment of vascular diseases, SIRT1 integrates the signaling transduction of STAT3 and HIF-1α, forming a balanced regulatory network; the disruption of this network represents an important mechanism underlying aberrant VSMCs activation [227].

The deacetylation modification of STAT3 by SIRT1 is bidirectionally regulated by multiple factors. WWP2, a HECT-type E3 ubiquitin ligase, is upregulated in Ang II-induced VSMCs models. WWP2 can interact with SIRT1 and STAT3 to form a ternary complex, reducing SIRT1-mediated deacetylation of STAT3 and promoting STAT3 acetylation and phosphorylation, which ultimately enhances VSMCs proliferation, migration, and phenotypic switching, thereby driving the pathogenesis and progression of hypertensive vascular lesions [228]. Conversely, Hcy can accelerate VSMCs proliferation, migration, and phenotypic switching by upregulating WWP2 expression to enhance the phosphorylation of SIRT1/STAT3 signaling, thereby accelerating the atherosclerotic process [22]. In contrast, Septin4 acts as a negative regulator of this pathway, forming a ternary complex with SIRT1 and STAT3 in PDGF-BB-induced human aortic VSMCs to promote SIRT1-mediated STAT3 deacetylation and dephosphorylation, thereby inhibiting VSMCs proliferation, migration, and phenotypic switching, and counteracting the progression of atherosclerosis [228].

### 4.4. Other Signaling Pathways

Beyond the aforementioned classical STAT3-related signaling pathways, STAT3 also functions as a downstream transcription factor node, forming an extensive cross-regulatory network with multiple signaling axes such as NF-κB, TGF-β/Smad, Wnt/β-catenin, and PI3K-Akt-mTOR to synergistically amplify inflammatory, proliferative, and phenotypic switching signals in VSMCs [229,230].

Cytokines such as TNF-α and IL-6 can simultaneously activate the IKK/NF-κB and JAK2/STAT3 pathways, while reactive oxygen species concurrently activate IKK and relieve the inhibition on JAK2 via oxidative inactivation of PTP1B, thereby initiating the synergistic activation of both pathways [231,232]. Activated NF-κB and STAT3 form enhanceosome complexes at promoters of pro-inflammatory genes (e.g., IL-6 and MCP-1) [232,233]. Furthermore, p65 physically interacts with the STAT3 SH2 domain [234], while shared co-activator p300/CBP acetylates both factors to amplify transcriptional potency [235,236]. Regarding NLRP3 inflammasome regulation, circACTA2 suppresses NLRP3-mediated inflammatory responses through its interaction with NF-κB, whereas STAT3 facilitates inflammasome activation by promoting the mitochondrial translocation of NLRP3, suggesting a functional division and interplay between the two factors during this process [17,183].

A pro-proliferative signaling axis exists between TGF-β and STAT3. Studies have revealed that the TGFβ-Stat3-FoxO1 axis facilitates VSMCs proliferation, whereas PPARγ, operating as an inhibitor of both this axis and TGFβ-Smad3/4 signaling, suppresses VSMCs phenotypic switching by physically interacting with Stat3 and Smad3 [237]. The intersection between the Wnt/β-catenin pathway and STAT3 plays a critical role in VC. By establishing an osteogenic model of human aortic smooth muscle cells, Zhang et al. demonstrated that moscatilin, a natural bibenzyl compound, can suppress STAT3 phosphorylation by binding to IL13RA2, thereby downregulating WNT3 expression and modulating the VSMCs phenotype to attenuate VC [238]. The cross-regulation between these signaling pathways and STAT3 is constrained by shared negative feedback mechanisms. SOCS3 can downregulate STAT3 signaling by inhibiting JAK2 activity, and it can also suppress the activation of the NF-κB pathway by interfering with the ubiquitination of IKKγ (also known as NEMO); this common negative feedback loop represents a critical node for maintaining the dynamic equilibrium of the signaling network and precisely modulating the VSMCs phenotype [230,239].

The PI3K-Akt-mTOR pathway and STAT3 cooperatively regulate VSMCs proliferation, migration, and phenotypic switching under various stimuli, constituting a critical signaling network in vascular remodeling diseases such as neointimal hyperplasia after vascular injury and atherosclerosis. Growth factors such as PDGF and VEGF serve as vital upstream signals driving this synergistic network. Hypoxia-induced HIF-1α facilitates the expression of PDGF and VEGF. On one hand, PDGF regulates cell cycle progression by activating the PI3K-Akt-mTOR pathway; on the other hand, it induces the expression of survival proteins such as cyclin D1 via STAT3 phosphorylation, with both actions jointly promoting VSMCs proliferation and migration while inhibiting apoptosis. Conversely, VEGF mediates VSMCs phenotypic switching by activating STAT3. The vasoactive factor Ang II likewise exerts a synergistic effect, simultaneously activating the PI3K-Akt-mTOR pathway and the JAK/STAT pathway via AT1R to cooperatively drive VSMCs proliferation. In addition to positive synergy, certain regulatory factors can simultaneously suppress both pathways. For instance, miR-145 inhibits VSMCs phenotypic switching, proliferation, and migration by suppressing PI3K-Akt-mTOR pathway activity, an effect that may be linked to the regulation of the STAT3-related signaling network [240]. Overexpression of the scavenger receptor SCARA5 can inhibit the phosphorylation of both STAT3 and the PI3K/AKT pathways, thereby suppressing cell proliferation and epithelial–mesenchymal transition; conversely, SCARA5 knockdown relieves the inhibition on the PDGF signaling pathway, promoting PDGF-BB-induced VSMCs proliferation and migration [241]. In the process of neointimal hyperplasia after vascular injury, the combination of sirolimus and rosuvastatin effectively suppresses neointimal hyperplasia through a stage-dependent regulation of both pathways. In the acute phase, it reduces the production of pro-inflammatory cytokines such as IL-6, blocking IL-6/STAT3 signaling activation to inhibit the initiation of VSMCs proliferation; in the chronic phase, it targets the Akt/mTOR/NF-κB/STAT3 signaling network to decrease the expression of Akt1, mTOR, p-STAT3, and NF-κB, thereby inhibiting MMP-9-mediated ECM remodeling and preventing VSMCs migration and phenotypic switching [242]. Furthermore, oxLDL-activated macrophage-derived exosomes can modulate the PI3K-Akt-mTOR pathway, thereby affecting the proliferation, invasion, and anti-apoptotic capacities of VSMCs [243].

## 5. STAT3 Inhibitors

Although a large amount of research work has been invested in discovering STAT3 inhibitors, this field remains one of the potential targets for therapeutic development. According to the mode of action of inhibitors, STAT3 inhibitors can be divided into two categories: direct inhibition and indirect inhibition [244]. In addition, a variety of natural products have been reported to exhibit STAT3 inhibitory activity [245].

### 5.1. Direct Targeting of STAT3

STAT3 direct targeting inhibitors inhibit downstream signaling transduction by directly binding to specific domains of the STAT3 protein and blocking its phosphorylation, dimerization, and DNA-binding activity [246]. During the process of STAT3 activation, the SH2 domain mediates the recognition of phosphorylated tyrosine residues by STAT3 and the occurrence of homodimerization, serving as a key molecular site for blocking STAT3 signal transduction. Currently, the vast majority of direct STAT3 inhibitors that have entered preclinical or clinical validation target the SH2 domain (Table 1). Below, their pharmacological effects in VSMCs will be introduced in sequence according to the development context and research characteristics of the inhibitors.

First, Stattic, as a representative of the most widely studied non-peptide small molecules, directly targets the SH2 domain of STAT3, thereby blocking its phosphorylation, dimerization, nuclear translocation, and DNA-binding capacity, ultimately specifically inhibiting the transcriptional activity of STAT3 [247]. Studies have found that by inhibiting STAT3 activity, Stattic can effectively reverse this pathological phenotypic transition of VSMCs from a contractile to a synthetic phenotype. It promotes the recovery of normal contractile function in VSMCs by downregulating the expression of synthetic markers (OPN, PCNA, vimentin) while upregulating the expression of contractile markers (SM-MHC, calponin, SM22α) [133]. Additionally, by inhibiting the activation of STAT3, Stattic indirectly suppresses the activation of the NLRP3 inflammasome and the cleavage of the GSDMD-N fragment, thereby reducing VSMCs pyroptosis, which in turn alleviates inflammatory responses and tissue injury [248]. In terms of regulating metabolic reprogramming, Stattic can interfere with the expression of key glycolytic enzymes, such as HK2, LDHA, and pyruvate dehydrogenase kinase 1 (PDK1), synergistically induced by HIF-1α/STAT3 by inhibiting STAT3 activity, thereby reducing glycolytic flux and achieving regulation over VSMCs metabolic reprogramming [249].

On the basis of Stattic, the novel inhibitor BP-1-102 has further optimized targeting efficiency and pharmacological activity. BP-1-102 is a potent novel STAT3 inhibitor designed to directly bind to the SH2 domain of the STAT3 protein, thereby preventing Tyr705 site phosphorylation, dimerization, and subsequent nuclear translocation of STAT3, ultimately inhibiting its activity as a transcription factor [250]. By inhibiting STAT3 activity, BP-1-102 arrests VSMCs in the G_0_/G_1_ phase to inhibit proliferation, upregulates contractile markers such as SM22α and α-SMA to restore the contractile phenotype, downregulates MMP-2 and MMP-9 to reduce migration, and simultaneously activates apoptotic programs to eliminate pathological VSMCs, thereby inhibiting the progression of CVDs [251].

Different from the two synthetic small molecules mentioned above, CAB (9H-Carbazol-3-yl 4-aminobenzoate), as a carbazole derivative, exhibits high selectivity and shows no significant inhibitory effect on a variety of other kinases. Studies have confirmed that CAB can directly bind to the SH2 domain of STAT3 and interact with Ile589, Arg609, and Glu612 residues. This binding specifically blocks the phosphorylation of the STAT3 Tyr705 site without affecting the Ser727 residue, while not inhibiting the activation of PDGFR-β, Akt, Erk1/2, p38, JAK2, or Src, showing a high degree of selectivity [252]. By inhibiting STAT3 activity, CAB downregulates the transcriptional level of its downstream target gene CIAPIN1, thereby weakening the activity of the CIAPIN1/JAK2/STAT3 positive feedback signaling axis. This process leads to the inhibition of KLF4-mediated VSMCs dedifferentiation, enhancement of CDKN1B-induced cell cycle arrest, and downregulation of MMP-9 expression, cooperatively blocking the vascular remodeling process from three levels: phenotypic transition, proliferation, and ECM degradation. In a rat carotid artery balloon injury-induced neointimal hyperplasia model, systemic administration of CAB significantly inhibits intimal hyperplasia. Studies have found that CIAPIN1 overexpression can partially reverse the inhibitory effects of CAB on VSMCs proliferation, migration, phenotypic transition, and intimal hyperplasia, confirming from a functional perspective that CIAPIN1 is a key downstream target for CAB to exert its pharmacological effects [139,252].

Another important class of inhibitors is based on the binaphthol sulfonamide scaffold, with representative compounds C188-9 and S3I-201 also exhibiting high affinity, though differences exist in their efficacy. C188-9 (TTI-101) is an orally bioavailable STAT3 SH2 domain inhibitor based on the binaphthol sulfonamide scaffold, competing for the STAT3 SH2 domain with extremely high affinity to prevent its tyrosine phosphorylation and homodimerization, thereby blocking STAT3-mediated target gene transcription. In a mouse model of cardiac fibrosis induced by Trypanosoma cruzi infection, TTI-101 can eliminate cardiac fibrosis and reduce serum levels of TGF-β and PDGF-D. However, this treatment simultaneously leads to increased myocardial inflammatory cell infiltration, accompanied by upregulated gene expression of the pro-inflammatory transcription factors STAT1 and NF-κB and elevated IL-6 protein levels, suggesting that STAT3 primarily plays a pro-fibrotic role during the process of cardiac fibrosis, whereas in inflammatory regulation, it may exert anti-inflammatory functions by inhibiting STAT1/NF-κB signaling [253] S3I-201 is another classic STAT3 SH2 domain inhibitor that can prevent STAT3 phosphorylation activation, dimerization, and DNA binding by binding to the STAT3 SH2 domai [254]. In Ang II-induced vascular dysfunction and hypertension models, S3I-201 can significantly inhibit superoxide anion production and the decline in endothelium-dependent vasodilation function in the mouse carotid artery, and prevent Ang II-induced blood pressure elevation, but does not affect Ang II-induced carotid hypertrophic remodeling [255]. In the Ang II-induced hypertensive nephropathy model, S3I-201 also exhibits potential therapeutic effects. Notably, the efficacy of S3I-201 is significantly lower than that of next-generation inhibitors such as C188-9, presenting certain limitations in its in vivo pharmacological applications [254,256].

In addition, STAT3 inhibitors from natural product sources can also target the SH2 domain, among which the curcumin analog HO-3867 exerts its inhibitory effect through covalent modification. HO-3867 can directly act on the SH2 domain of STAT3, blocking STAT3 phosphorylation through covalent modification of the cysteine residue Cys259 within the SH2 domain [257]. By inhibiting STAT3 activity, HO-3867 can prevent or reverse phenotypic transition, restoring VSMCs to a quiescent contractile state. This may be achieved by upregulating myogenic genes (such as MYOCD, SRF, ACTA2, CNN1) and downregulating synthetic markers (such as osteopontin, Ki-67) [136]. Recent studies indicate that hypoxia-inducible factor 1-α (HIF-1α) affects the phenotypic transition of VSMCs by regulating metabolic status, and a potential synergy exists between STAT3 and HIF-1α. By inhibiting STAT3 activity, HO-3867 may indirectly affect the HIF-1α pathway, thereby further regulating the phenotypic transition of VSMCs [258].

I3MO (Indirubin-3′-monoxime) is a semi-synthetic derivative originating from indirubin, the active component of the traditional Chinese medicine formula Danggui Longhui Wan. It was initially identified as a CDK inhibitor, but subsequent studies have confirmed that it exerts anti-proliferative effects in VSMCs primarily through specific blockade of STAT3 signaling [259]. In vivo studies found that intraperitoneal injection of I3MO significantly inhibits neointima formation in a mouse femoral artery cuff injury model, markedly reducing the neointima/media ratio, accompanied by a decrease in STAT3 phosphorylation levels and a reduction in the number of Ki67-positive proliferating cells. Meanwhile, in vitro experiments confirmed that I3MO arrests PDGF-BB, IFN-γ, and thrombin-induced VSMCs in the G_0_/G_1_ phase in a dose-dependent manner, thereby inhibiting their proliferation; its mechanism of action may be related to the fact that I3MO has no inhibitory effect on tyrosine or serine/threonine kinases such as Akt, Erk1/2, and p38 MAPK, but can specifically block STAT3 Tyr705 phosphorylation induced by these three different stimuli [259]. In addition, some scholars have further revealed a novel upstream inhibitory pathway of I3MO that is distinct from directly binding to the SH2 domain, namely that I3MO can directly inhibit 12/15-lipoxygenase (12/15-LO) activity and block PDGF-BB-induced ROS/15(S)-HETE production and downstream STAT3 phosphorylation, thereby achieving negative regulation of STAT3 at a more upstream level of the signaling pathway [260]. Moreover, recent studies reveal that PDGF-BB stimulation can induce aerobic glycolysis and upregulate HK2 expression in VSMCs, and I3MO inhibits glucose metabolic reprogramming by preventing STAT3-mediated transcriptional upregulation of HK2, thereby suppressing VSMCs migration [110].

### 5.2. Indirect Targeting of STAT3

Indirect STAT3 inhibitors refer to a class of compounds or biological molecules that do not directly bind to the STAT3 protein, but instead indirectly reduce STAT3 phosphorylation, nuclear translocation, DNA-binding capacity, and transcriptional activity by interfering with upstream activation signals of STAT3, promoting the expression of STAT3 negative regulators, enhancing the activity of STAT3 negative regulators, or accelerating the degradation of the STAT3 protein [261]. These inhibitors indirectly inhibit STAT3 activity by modulating upstream kinases of STAT3 (such as JAKs, Src family kinases, etc.) or downstream pathways, or by affecting the negative regulators of STAT3 (including SOCS3, PIAS3, etc.) [262]. Indirect STAT3 inhibitors regulate processes such as pathological phenotypic transition, proliferation, migration, metabolic reprogramming, as well as pyroptosis and apoptosis of VSMCs through multiple mechanisms, which are highly dependent on the cellular microenvironment and finely regulated by local signals such as inflammatory factors and co-activators [263] (Table 2).

AG490 is a selective JAK2 inhibitor that blocks STAT3 phosphorylation by inhibiting JAK2 kinase activity, thereby indirectly inhibiting the STAT3 signaling pathway. In a rat carotid artery balloon injury model, local administration of AG490 to the injured vessel can inhibit the constitutive phosphorylation of STAT3, suppress the replication of VSMCs in the neointima, and reduce neointima formation [264]. In a rat common carotid artery end-to-end anastomosis model, AG490 downregulates the expressions of p-JAK2, p-STAT3, and PCNA in the vascular wall, reduces postoperative vascular wall proliferation, inhibits the phenotypic transition of VSMCs toward the synthetic phenotype, and upregulates the expressions of contractile markers such as α-SMA and SM22α [265]. In vitro, AG490 can block PDGF-BB-induced STAT3 phosphorylation and DNA-binding activity in VSMCs, inhibiting cell motility [266]. In human aortic smooth muscle cells, AG490 can completely abolish PDGF-BB-stimulated cell proliferation [267]. AG490 can also block the osteogenic differentiation of VSMCs and the induction of RUNX2 by inhibiting the JAK2/STAT3 pathway, alleviating calcium deposition in a diabetic VC model [268].

**Table 1:** Direct STAT3 inhibitors in VSMCs.

Inhibitor Name	Chemical Structure	Target/Mechanism of Action	Primary Effects on VSMCs	Key Experimental Evidence/Model	References
Stattic	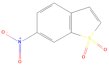	STAT3 SH2 domain; blocks Tyr705 phosphorylation, dimerization, and nuclear translocation.	Reverses synthetic phenotype (suppresses OPN/PCNA; restores SM22α/SM-MHC); inhibits NLRP3/GSDMD pyroptosis; attenuates HK2/LDHA-mediated glycolysis.	Rat carotid artery balloon injury; in vitro VSMC pyroptosis; HIF-1α/STAT3 metabolic assays.	[133,245,246,247]
BP-1-102	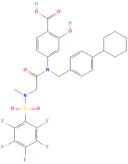	STAT3 SH2 domain; prevents Tyr705 phosphorylation, dimerization, and nuclear entry.	Induces G_0_/G_1_ cell cycle arrest and apoptosis; restores contractile markers (SM22α, α-SMA); downregulates MMP-2/9 migration.	VSMC proliferation, migration, and phenotypic switching assays.	[248,249]
CAB	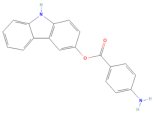	Selectively binds STAT3 SH2 domain (Ile589/Arg609/Glu612); blocks Tyr705 phosphorylation.	Reverses KLF4-driven dedifferentiation via CIAPIN1 downregulation; induces CDKN1B-mediated cell cycle arrest; downregulates MMP-9.	Rat carotid balloon injury; CIAPIN1 rescue assays.	[139,250]
C188-9	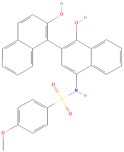	High-affinity competitive STAT3 SH2 domain inhibitor; blocks tyrosine phosphorylation.	Suppresses cardiac fibrosis (reduces TGF-β and PDGF-D); modulates STAT1/NF-κB inflammatory signaling.	Trypanosoma cruzi-induced cardiac fibrosis mouse model.	[251]
S3I-201	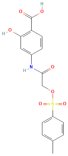	STAT3 SH2 domain; inhibits phosphorylation, dimerization, and DNA binding.	Suppresses Ang II-induced superoxide generation; restores endothelial vasodilation and lowers blood pressure; improves hypertensive nephropathy.	Ang II-induced hypertensive mouse model; hypertensive nephropathy assays.	[252,253,254]
HO-3867	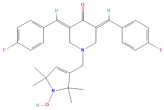	Covalently modifies Cys259 residue in STAT3 SH2 domain; inhibits phosphorylation.	Restores contractile phenotype (upregulates MYOCD/ACTA2; downregulates OPN/Ki-67); reverses HIF-1α-driven metabolic alterations.	In vitro VSMC phenotypic switching; HIF-1α synergistic metabolic regulation.	[136,255,256]
I3MO	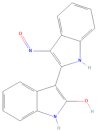	Inhibits 12/15-LO activity; blocks upstream ROS/15(S)-HETE signaling and STAT3 activation.	Suppresses neointima formation; induces G_0_/G_1_ arrest; selectively blocks STAT3 Tyr705 phosphorylation; halts HK2-dependent glycolysis.	Mouse femoral artery cuff injury; in vitro PDGF-BB/IFN-γ assays.	[110,257,258]

Based on the structural optimization of AG490, WP1066, as a novel JAK2 and STAT3 inhibitor, indirectly blocks the STAT3 signaling pathway by inhibiting JAK2 kinase activity and its downstream STAT3 phosphorylation. In vivo, Daniel et al. confirmed in a wire-injured mouse femoral artery model that WP1066, applied locally around the dilated artery via a temperature-sensitive Pluronic F-127 gel, can eliminate STAT3 phosphorylation, inhibit neointimal cell proliferation, increase the proportion of apoptotic cells, significantly decrease the neointimal area 3 weeks after injury, and does not affect the local recruitment of circulating inflammatory cells [269]. In vitro, WP1066 dose-dependently blocks PDGF-BB-stimulated STAT3 phosphorylation in rat aortic smooth muscle cells, inhibiting STAT3 nuclear translocation and the transcriptional activation of downstream target genes cyclin D1 and survivin, thereby inhibiting VSMCs proliferation and migration [269]. In human umbilical artery smooth muscle cells, WP1066 further inhibits Ang II-induced activation of the JAK2/STAT3/SOCS3 signaling pathway, abandoning oxidative stress and cell proliferation [270]. Additionally, in a study on irisin regulating VSMCs phenotypic transition, WP1066 was used as a STAT3 inhibitor tool compound, revealing the critical role of STAT3 in PDGF-BB-induced VSMCs phenotypic transition [271].

Different from the aforementioned selective JAK2 inhibitors, ruxolitinib is an inhibitor that simultaneously targets JAK1 and JAK2, indirectly inhibiting the STAT3 signaling pathway by blocking upstream JAK kinase-mediated STAT3 phosphorylation. Ruxolitinib plays a key role in regulating various biological processes of VSMCs, such as phenotypic transition, proliferation, migration, apoptosis, pyroptosis, and metabolic reprogramming [272,273]. In a rat carotid artery balloon injury model, ruxolitinib can significantly inhibit STAT3 phosphorylation and Cyclin D1 expression, effectively reducing neointimal hyperplasia [274]. This effect originates from ruxolitinib inhibiting STAT3 phosphorylation, preventing its dimerization and nuclear translocation, thereby blocking the transcriptional driving of STAT3 on the cell cycle protein Cyclin D1 and the anti-apoptotic protein Bcl-xL [275]. Meanwhile, ruxolitinib blocks the phenotypic transition of VSMCs from a contractile to a synthetic phenotype by downregulating KLF4 and myocardin (MYOCD) and inhibiting the expression of SRF-dependent contractile genes (including ACTA2, CNN1, and MYH11). Additionally, ruxolitinib can also interfere with NF-κB/STAT3 signaling crosstalk, inhibit the initiation of the NLRP3 inflammasome, and reduce Gasdermin D (GSDMD) cleavage, indirectly inhibiting caspase-1 activation, thereby alleviating VSMCs pyroptosis [276].

Tofacitinib mainly targets JAK1 and JAK3, indirectly inhibiting STAT3 phosphorylation and subsequent signaling transduction by blocking the upstream JAK/STAT signaling cascade. In an ApoE−/− mouse model of atherosclerosis, intragastric administration of tofacitinib can inhibit JAK/STAT signaling transduction, lower STAT3 phosphorylation levels, and reduce atherosclerotic plaque area at the aortic root and foam cell formation, the molecular mechanism of which involves downregulating the expressions of pro-inflammatory cytokines IL-6 and TNF-α and upregulating ABCA1 expression [277]. In an MRL/lpr lupus mouse model, tofacitinib improves endothelium-dependent vasodilation function by inhibiting the JAK/STAT3 signaling pathway, demonstrating a vasculoprotective effect independent of its immunomodulatory activity [278]. Additionally, Parra-Izquierdo et al. found that IFN-α stimulation of human aortic valve interstitial cells can promote calcified nodule formation by activating JAK/STAT signaling, and tofacitinib effectively inhibits the calcification process by blocking downstream STAT3 phosphorylation of this pathway [279].

Urantide, as a selective urotensin II receptor antagonist, exerts its therapeutic effects on CVDs, especially atherosclerosis, primarily by modulating the JAK2/STAT3 signaling pathway to affect multiple biological processes of VSMCs [280]. In an atherosclerotic rat model, Urantide treatment can significantly reduce the expression of UII and its receptor GPR14, while downregulating the levels of phosphorylated JAK2 and phosphorylated STAT3 by inhibiting the JAK2/STAT3 signaling pathway [133]. This indicates that Urantide inhibits the activation of the JAK2/STAT3 signaling pathway by regulating the UII/UT system, thereby suppressing the aberrant proliferation and migration of VSMCs. Additionally, Urantide can regulate collagen metabolism in the hearts of atherosclerotic rats and alleviate myocardial fibrosis by inhibiting the JAK2/STAT3 pathway. Specifically, Urantide reduces the expression of MMP2 and increases the expression of tissue inhibitor of metalloproteinases 2 (TIMP2), thereby regulating the balance between collagen synthesis and degradation. This mechanism of action is closely related to the downregulation of the phosphorylation levels of JAK2 and STAT3 by Urantide [280].

### 5.3. Natural Products as STAT3 Inhibitors

STAT3 natural product inhibitors refer to small-molecule compounds derived from natural resources such as plants, microorganisms, or marine organisms, which can specifically regulate the STAT3 signaling pathway through various mechanisms. Their direct inhibitory mechanism involves directly binding to the STAT3 protein and blocking its Tyr705 site phosphorylation, dimerization, nuclear translocation, and DNA-binding activity, thereby inhibiting the transcription of downstream target genes [281]. The indirect inhibitory mechanism reduces the activation level of STAT3 by interfering with the activity of upstream kinases of STAT3 or upregulating negative regulators such as PTPs and SOCS3 [282] (Table 3).

Curcumin originates from the rhizome of the Zingiberaceae plant Curcuma longa and is the primary active component of the traditional Chinese medicine Jianghuang. It belongs to natural polyphenolic compounds and possesses various pharmacological activities such as anti-inflammatory, antioxidant, lipid-regulating, and anti-atherosclerotic effects [283]. Multiple clinical meta-analysis studies have shown that curcumin supplementation can significantly reduce diastolic blood pressure, pulse wave velocity, and vascular cell adhesion molecule-1 levels, while increasing flow-mediated dilation, thereby improving vascular endothelial function [284]. The inhibition of STAT3 by curcumin involves dual direct and indirect mechanisms simultaneously. At the direct inhibition level, curcumin can directly bind to the SH2 domain of STAT3, hindering the tyrosine phosphorylation of the STAT3 Tyr705 site, thereby inhibiting STAT3 dimerization and nuclear translocation, and blocking its DNA-binding activity and downstream gene transcription [285]. At the indirect inhibition level, curcumin can inhibit the activities of upstream tyrosine kinases such as JAK, Src, and EGFR, reducing STAT3 phosphorylation from the source [286]. Distinct from simply inhibiting upstream kinases, curcumin can also promote the activities of PTPs such as SHP-1, SHP-2, and PTP1B, accelerating the dephosphorylation of already phosphorylated STAT3 and prompting its inactivation. Studies have found that curcumin can significantly inhibit neointima formation, reduce collagen accumulation and PDGF receptor phosphorylation in carotid artery balloon injury model rats, and concentration-dependently inhibit PDGF-BB-induced migration, proliferation, and collagen synthesis of VSMCs [287]. Additionally, curcumin can upregulate the expression of the contractile marker SM22α and reverse the pathological phenotypic transition of VSMCs by activating myocardin expression [288].

**Table 2:** Indirect STAT3 inhibitors in VSMCs.

Inhibitor Name	Chemical Structure	Target/Mechanism of Action	Primary Effects on VSMCs	Key Experimental Evidence/Model	References
AG490	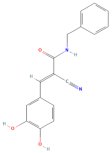	Selective JAK2 kinase inhibitor; suppresses downstream STAT3 phosphorylation.	Abolishes STAT3 phosphorylation and neointimal proliferation; upregulates α-SMA and SM22α; inhibits RUNX2-mediated calcification.	Rat carotid balloon injury; end-to-end anastomosis; diabetic VC model.	[262,263,264,265,266]
WP1066	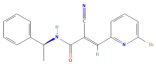	Dual JAK2/STAT3 inhibitor; blocks JAK2 activity and STAT3 phosphorylation.	Eliminates p-STAT3 and triggers apoptosis; downregulates cyclin D1 and survivin; attenuates Ang II-induced oxidative stress.	Mouse femoral wire injury (local Pluronic gel delivery); Ang II-induced VSMC assays.	[267,268,269]
Ruxolitinib	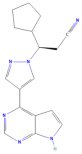	JAK1/JAK2 kinase inhibitor; blocks upstream phosphorylation cascades.	Inhibits Cyclin D1/Bcl-xL to halt cell cycle; restores contractile state via KLF4/MYOCD axis; suppresses NLRP3/GSDMD pyroptosis.	Rat carotid balloon injury; NF-κB/STAT3 crosstalk; pyroptosis regulation.	[270,271,272,273,274]
Tofacitinib	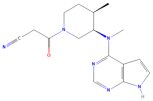	JAK1/JAK3 kinase inhibitor; intercepts upstream JAK/STAT signaling.	Reduces aortic plaque area and foam cells via ABCA1 upregulation; improves endothelial vasodilation; inhibits valvular calcific nodules.	ApoE−/− mice; MRL/lpr lupus model; human aortic valve interstitial cells.	[275,276,277]
Urantide	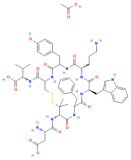	UII/UT (GPR14) receptor antagonist; downregulates the JAK2/STAT3 axis.	Reduces p-JAK2 and p-STAT3 levels; suppresses abnormal proliferation and migration; rebalances MMP-2/TIMP-2 to alleviate fibrosis.	Atherosclerotic rat model; cardiac collagen metabolism analysis.	[133,278]

Resveratrol is a natural polyphenolic compound primarily derived from plants such as Polygonum cuspidatum, grapes, and peanuts, possessing various pharmacological activities including anti-inflammatory, antioxidant, anti-tumor, and cardiovascular protective effects [289]. Its inhibitory effect on the STAT3 signaling pathway also involves multiple molecular levels, exhibiting a regulatory pattern similar to that of curcumin [290]. Resveratrol can inhibit the tyrosine phosphorylation of the STAT3 Tyr705 site and can directly or indirectly inhibit the activities of STAT3 upstream kinases such as JAK2 and c-Src, reducing the phosphorylation of STAT3 [291]. Resveratrol exerts multiple pharmacological effects by inhibiting the STAT3 signaling pathway. In VC, resveratrol interferes with the phenotypic transition of VSMCs from a contractile to a synthetic phenotype by inhibiting the STAT3 pathway [292]. Meanwhile, resveratrol downregulates the expressions of cyclin D1 and CDK4 and upregulates the expressions of p21 and p27 by inhibiting the STAT3 pathway, inducing VSMCs arrest in the G_0_/G_1_ phase and inhibiting cell proliferation [293]. Additionally, resveratrol can suppress NF-κB-driven transcription of the NLRP3 inflammasome and pro-IL-1β and reduce the cleavage of Gasdermin D by inhibiting the STAT3 signaling pathway, thereby inhibiting VSMCs pyroptosis [248].

Epigallocatechin-3-gallate (EGCG) is one of the most abundant catechin polyphenolic components in green tea (Camellia sinensis), possessing various pharmacological activities such as antioxidant, anti-inflammatory, anti-atherosclerotic, and cardiovascular protective effects. The regulation of the STAT3 signaling pathway in VSMCs by EGCG features the dual characteristics of both direct binding and indirect inhibition. EGCG can significantly block the binding of STAT3 to phosphorylated peptides at micromolar concentrations. Molecular docking results show that EGCG has a strong interaction with Arg-609, a key residue in the STAT3 SH2 domain involved in binding phosphorylated peptides, indicating that EGCG can directly interfere with the function of the STAT3 SH2 domain, thereby inhibiting STAT3 dimerization and downstream signaling transduction [294]. Additionally, EGCG can directly inhibit the kinase activity of JAK2, thereby blocking STAT3 phosphorylation [295]. In primarily cultured Sprague–Dawley rat thoracic aortic VSMCs, EGCG can significantly downregulate the protein levels of JAK2, STAT3, and the phosphorylation level of STAT3 upregulated by PDGF-BB stimulation, and its inhibitory effect is comparable to that of an equal concentration of the JAK2-specific inhibitor AG490; moreover, both EGCG and AG490 can significantly inhibit PDGF-BB-induced proliferation and migration of VSMCs. EGCG also demonstrates efficacy in inhibiting neointima formation in in vivo vascular injury models. In a rat carotid artery balloon injury model, EGCG intervention can significantly reduce the neointimal area and lower the neointima/media area ratio [296]. Furthermore, green tea catechins can effectively inhibit VSMCs proliferation and prevent neointima formation in a rat carotid artery injury model after local delivery, and EGCG, as the major active monomer of green tea catechins, exhibits similar effects [297].

Cryptotanshinone (CT) is derived from the root of Salvia miltiorrhiza of the Lamiaceae family and is the primary lipid-soluble active component of the traditional Chinese medicine Danshen. It belongs to diterpenoid quinone compounds and possesses multiple pharmacological activities including anti-inflammatory, antioxidant, anti-atherosclerotic, and anti-tumor effects [298]. The inhibition of STAT3 by cryptotanshinone involves dual direct and indirect mechanisms. Cryptotanshinone can rapidly inhibit STAT3 Tyr705 phosphorylation through interaction with the SH2 domain of STAT3 and significantly suppress its nuclear translocation by blocking STAT3 dimerization [299]. Furthermore, cryptotanshinone can significantly inhibit JAK2 phosphorylation without affecting the activities of upstream kinases such as c-Src and EGFR, thereby selectively blocking the JAK2/STAT3 signaling cascade. In vivo, cryptotanshinone has demonstrated effects in inhibiting STAT3 signaling and pathological vascular remodeling in aortic aneurysm, AD, and VC models. In an sST2-induced aortic aneurysm model, cryptotanshinone effectively reverses maladaptive aortic remodeling in vivo by inhibiting STAT3 phosphorylation and blocking the sST2-STAT3-CXCL1 signaling axis. In an ApoE−/− mouse model of atherosclerosis, oral administration of cryptotanshinone can significantly reduce the atherosclerotic plaque area and enhance plaque stability, the mechanism of which involves inhibiting the expressions of LOX-1 and MMP-9 as well as reactive oxygen species generation and NF-κB activation [300]. In a CKD rat VC model, cryptotanshinone partially blocks EPO-induced calcium deposition in VSMCs by inhibiting the JAK2/STAT3/BMP-2 axis [301]. In vitro, cryptotanshinone concentration-dependently inhibits TNF-α-induced MMP-9 production and cell migration in human aortic smooth muscle cells, the molecular mechanism of which involves downregulating the nuclear translocations of NF-κB and AP-1 [302]. Additionally, in primary rat VSMCs, cryptotanshinone as a STAT3 inhibitor can partially block EPO-induced activation of the JAK2/STAT3/BMP-2 axis and calcium deposition [301].

Quercetin is a natural flavonoid compound primarily present in various Chinese herbal medicines such as Sophora japonica, ginkgo, Schisandra chinensis, and Houttuynia cordata, possessing multiple pharmacological activities including antioxidant, anti-inflammatory, anti-tumor, lipid-regulating, hypoglycemic, and cardiovascular protective effects [303]. Meta-analyses of randomized controlled trials in clinical settings show that quercetin supplementation can significantly lower systolic blood pressure and improve vascular endothelial function in patients with metabolic syndrome [304]. The inhibition of STAT3 by quercetin exhibits typical characteristics of indirect inhibition, with its core target being the upstream JAK2 kinase rather than the STAT3 protein itself. Quercetin can effectively block the binding of Ang II to AT1R and, by inhibiting JAK2 phosphorylation, block the phosphorylation modification of STAT3 by JAK2, thereby inhibiting the entire process of STAT3 activation, dimerization, and nuclear translocation [305]. Additionally, quercetin can block KLF4-mediated phenotypic transition of VSMCs into macrophage-like cells by inhibiting the JAK2/STAT3 pathway, reducing intracellular lipid uptake and lipid accumulation, effectively preventing the formation of VSMC-derived foam cells [306]. Quercetin demonstrates effects in inhibiting vascular remodeling in both vascular restenosis and atherosclerosis models. In a rat abdominal aortic balloon injury model, oral administration of quercetin can significantly decrease the neointimal area, reduce the neointima/media ratio, and inhibit the expressions of PCNA, PDGF-BB, b-FGF, and TGF-β as well as collagen deposition in the neointima [307]. In a high-fat diet-induced atherosclerotic model in ApoE−/− mice, quercetin can attenuate the aortic atherosclerotic plaque area and lipid deposition, and significantly downregulate the expressions of p-JAK2, p-STAT3, and KLF4 in foam cell aggregation areas [218]. Concurrently, quercetin concentration-dependently inhibits Ang II-induced proliferation of VSMCs, significantly reducing cell viability and cell number, inhibiting PCNA expression, and blocking the activation of the JAK2/STAT3 pathway by decreasing the p-JAK2/JAK2 and p-STAT3/STAT3 ratios [308].

Berberine is an isoquinoline alkaloid extracted from the rhizome of Coptis chinensis of the Ranunculaceae family and is the major active component of the traditional Chinese medicine Huanglian, possessing various pharmacological activities including anti-inflammatory, antibacterial, antioxidant, hypoglycemic, lipid-regulating, and anti-atherosclerotic effects [309]. The inhibition of STAT3 by berberine exhibits typical characteristics of indirect inhibition, with its core target being the upstream JAK2 kinase. Studies found that berberine blocks the phosphorylation modification of the STAT3 Tyr705 site by JAK2 by downregulating JAK2 expression and its kinase activity, thereby inhibiting STAT3 dimerization and nuclear translocation, and ultimately blocking the transcriptional activation of downstream target genes. The study further utilized the STAT3-specific activator Colivelin for reverse validation, confirming that Colivelin can significantly weaken the inhibitory effect of berberine on the JAK2/STAT3 pathway, aggravating VSMCs phenotypic transition, proliferation, and migration, which confirms from the perspective of functional recovery that the [145]. Additionally, chronic administration of berberine can effectively inhibit neointima formation, lower the neointima/media ratio [310], and significantly inhibit Ang II- and HB-EGF-induced proliferation and migration of rat VSMCs [145].

**Table 3:** Natural STAT3 inhibitors in VSMCs.

Natural Product	Compound Class	Mechanism of STAT3 Regulation	Type of Regulation	Functional Validation in VSMCs	References
Curcumin	Polyphenols	Binds STAT3 SH2 domain (Cys259); inhibits JAK/Src/EGFR kinases; activates SHP-1/2 and PTP1B dephosphorylation.	Direct/Indirect	Upregulates SM22α and activates myocardin to reverse phenotypic switching; inhibits PDGF-BB-induced proliferation and migration; reduces neointimal collagen deposition.	[279,280,281,282,283,284]
Resveratrol	Polyphenols	Inhibits STAT3 Tyr705 phosphorylation; suppresses upstream JAK2/c-Src kinases; downregulates NF-κB/NLRP3 signaling.	Direct/Indirect	Downregulates cyclin D1/CDK4 and upregulates p21/p27 to induce G_0_/G_1_ arrest; intervenes in calcification; inhibits GSDMD pyroptosis.	[243,285,286,287,288,289]
EGCG	Catechins	Engages Arg-609 in the STAT3 SH2 domain; directly inhibits JAK2 kinase activity.	Direct/Indirect	Downregulates p-STAT3 (comparable to AG490); suppresses PDGF-BB-induced proliferation and migration; lowers in vivo neointima/media ratio.	[290,291,292,293]
Cryptotanshinone	Diterpenoid quinones	Binds STAT3 SH2 domain; selectively inhibits JAK2 phosphorylation without affecting c-Src/EGFR.	Direct/Indirect	Downregulates NF-κB/AP-1-driven MMP-9 and cell motility; blocks EPO-induced calcification; reduces plaque area and enhances stability in ApoE−/− mice.	[294,295,296,297,298]
Quercetin	Flavonoids	Blocks AT1R-JAK2 coupling, preventing JAK2-mediated phosphorylation of STAT3 Tyr705.	Indirect	Suppresses Ang II-induced proliferation (lowers PCNA); blocks KLF4-driven macrophage-like foam cell formation; attenuates neointima and lipid accumulation.	[213,299,300,301,302,303,304]
Berberine	Isoquinoline alkaloids	Downregulates JAK2 expression and kinase activity, preventing STAT3 Tyr705 phosphorylation and nuclear translocation.	Indirect	Inhibits Ang II/HB-EGF-induced VSMC migration; restores contractile markers; target selectivity functionally validated by Colivelin rescue.	[141,305,306]

### 5.4. Clinical Translation and Investigational Drugs

Translating the extensive preclinical mechanistic insights on the STAT3 signaling axis into cardiovascular therapeutics presents substantial translational opportunities as well as distinct challenges [224,225]. Currently, clinical efforts targeting this signaling network are advancing through two complementary pathways, where one focuses on evaluating the vascular protective effects of established pharmacotherapies, while the other involves developing emerging targeted investigational candidates across clinical trials (Table 4).

Among the indirect kinase inhibitors detailed in previous sections, FDA-approved small-molecule JAK inhibitors represent the most clinically accessible agents that modulate downstream STAT3 activation [82,274]. Clinical cohort observations and post-hoc analyses in patients with systemic inflammatory conditions have confirmed that tofacitinib and ruxolitinib substantially alleviate vascular inflammatory burdens, restore endothelium-dependent vasodilation, and halt carotid intima-media thickening [272,278]. In parallel, among direct STAT3-targeting molecules, the orally bioavailable SH2 domain inhibitor TTI-101 (formerly C188-9) has successfully completed phase 1 dose-escalation evaluations (NCT03195699), demonstrating favorable safety profiles, linear pharmacokinetics, and verified on-target STAT3 engagement, thereby providing a foundational clinical benchmark for direct small-molecule STAT3 inhibition in humans [253].

Beyond these established agents, novel investigational therapeutics tailored to intercept the pathway at distinct molecular tiers have entered dedicated cardiovascular and translational clinical trials. At the upstream ligand level, direct neutralization of IL-6 by the fully humanized monoclonal antibody ziltivekimab markedly suppressed high-sensitivity C-reactive protein and atherogenic inflammatory biomarkers in patients with atherosclerosis and chronic kidney disease during the phase 2 RESCUE trial [311], which established the clinical premise for the large-scale phase 3 ZEUS cardiovascular outcomes trial [312]. At the receptor level, the IL-6 receptor antagonist tocilizumab demonstrated cardioprotective efficacy by attenuating systemic vascular inflammation, reducing troponin release, and limiting myocardial ischemic damage in randomized trials of acute coronary syndromes, including NSTEMI [313] and the ASSAIL-MI trial in STEMI [314]. Furthermore, to bypass the historical challenge of targeting undruggable transcription factors with conventional chemical compounds, nucleic acid-based modalities have achieved clinical proof-of-concept. Specifically, the next-generation antisense oligonucleotide danvatirsen (AZD9150) demonstrated robust on-target STAT3 mRNA downregulation and acceptable clinical tolerability in both monotherapy [315] and combination regimens (NCT02983578, NCT05986240) [316].

Nevertheless, clinical translation of systemic STAT3 inhibitors for cardiovascular indications remains constrained by on-target toxicities, including cytopenias, impaired immune defenses, and potential disturbance of baseline cardiomyocyte survival pathways [116,224]. To overcome these translational hurdles, localized and cell-selective delivery modalities are increasingly gaining momentum. Integrating STAT3 inhibitors into drug-eluting stents or balloons, alongside engineering biomimetic nanocarriers functionalized specifically for synthetic VSMCs, represents a promising horizon to suppress pathological vascular remodeling locally while minimizing systemic adverse events [64,242].

## 6. Perspectives

In recent years, substantial progress has been made in unraveling the intricate molecular networks through which STAT3 regulates VSMC plasticity and vascular wall homeostasis. The research paradigm has progressively transitioned from isolated, linear signaling cascades to multifaceted regulatory dimensions encompassing chronic inflammation, oxidative stress, epigenetic modifications, cellular metabolism, and cell fate decisions. Compelling preclinical evidence has established STAT3 as a pivotal molecular node driving the onset and progression of major cardiovascular disorders, including atherosclerosis, post-angioplasty restenosis, AD, and VC. Concurrently, an expanding repertoire of direct structural-domain inhibitors, upstream kinase antagonists, bioactive natural products, and clinical-stage investigational candidates have yielded encouraging experimental and preliminary translational outcomes. Nevertheless, establishing a spatiotemporally resolved understanding of STAT3 signaling dynamics in diseased vascular walls and bridging the divide between preclinical proof-of-concept and clinical application remain major frontiers in cardiovascular medicine. To accelerate the translation of these discoveries into actionable clinical interventions, several key scientific challenges and strategic directions warrant prioritized exploration.

Under the context of vascular cellular heterogeneity, the fine-tuned regulatory mechanisms of STAT3 within distinct VSMC subpopulations require systematic dissection. Lineage-tracing studies, single-cell RNA sequencing, and spatial multi-omics have unequivocally demonstrated that VSMCs do not represent a homogeneous contractile population. Instead, they adopt multiple specialized phenotypes during vascular lesion evolution, giving rise to distinct synthetic, inflammatory, macrophage-like, osteogenic, and senescent functional subsets that execute divergent or even opposing roles in different vascular diseases. Whether STAT3 activation intensity, post-translational modification profiles, and downstream transcriptional regulons differ significantly among these specific cellular states remains largely undefined. Future research should leverage high-resolution spatial transcriptomics and single-cell epigenomic mapping to trace STAT3-driven fate trajectories across distinct vascular microenvironmental niches, thereby establishing a cellular-resolution atlas of STAT3 signaling in vascular remodeling.

Beyond cellular heterogeneity, the cooperative interplay between canonical transcriptional regulation and non-canonical subcellular functions of STAT3 warrants deeper mechanistic interrogation. While the vast majority of current drug discovery programs focus on Tyr705 phosphorylation-mediated dimerization and nuclear transactivation, non-canonical STAT3 actions driven by Ser727 phosphorylation or specific organelle targeting—such as mitochondrial mitoSTAT3 and endoplasmic reticulum ERSTAT3—play vital homeostatic roles in maintaining electron transport chain activity, mitochondrial membrane potential, redox equilibrium, and inter-organelle calcium fluxes. These non-canonical actions are intimately linked to VSMC metabolic adaptation, oxidative stress tolerance, and cytoprotection against ischemic or mechanical damage. Future studies must elucidate the functional partition and dynamic equilibrium between nuclear and extranuclear STAT3 pools under baseline versus stressed states, avoiding an oversimplified view that restricts STAT3 function solely to nuclear gene transcription.

Furthermore, the context-dependent, bidirectional regulatory capacity of STAT3 across different stages of vascular diseases represents a fundamental conceptual issue. Although excessive and sustained STAT3 activation accelerates pathological vessel wall expansion by promoting abnormal VSMC proliferation, migration, matrix degradation, and pyroptotic inflammation, moderate or transient STAT3 activation provides essential physiological and reparative signals. For instance, in early vascular injury and aortic dissection models, basal STAT3 activation preserves vascular tensile strength by coordinating adaptive collagen deposition and supporting baseline survival pathways. This functional duality implies that global and unselective ablation of STAT3 activity may disrupt endogenous vascular repair mechanisms. Identifying the precise molecular switches that dictate the functional transition of STAT3 from homeostatic defense to maladaptive remodeling across acute versus chronic disease phases will provide the conceptual foundation for defining stage-specific therapeutic windows.

In parallel, the complex cross-regulatory networks connecting STAT3 with parallel signaling pathways and immunometabolic reprogramming require holistic, systems-level investigation. In diseased vessels, STAT3 intersects with multiple signaling cascades including NF-κB, TGF-β/Smad, Wnt/β-catenin, and PI3K-Akt-mTOR through shared co-activators, reciprocal transcriptional enhanceosomes, and common negative feedback loops such as SOCS3. Decoding the hierarchical organization and dominant driving nodes within these interconnected networks will benefit from high-throughput phosphoproteomics, chromatin accessibility profiling, and artificial intelligence-assisted network biology. Moreover, because phenotypic switching in VSMCs is tightly coupled with metabolic shifts toward aerobic glycolysis, altered glutaminolysis, and disrupted lipid handling, combining stable isotope-resolved metabolomics with functional flux assays will uncover STAT3-dependent metabolic vulnerabilities tailored to specific disease microenvironments.

Finally, the clinical translation of STAT3-targeted pharmacological interventions requires innovative drug delivery modalities to overcome intrinsic safety and efficacy bottlenecks. Because STAT3 executes broad physiological functions across normal cardiovascular and immune tissues, traditional systemic pan-inhibition risks triggering unacceptable on-target toxicities, including cytopenias, impaired host defenses, and the loss of baseline cardiomyocyte survival signaling. To circumvent these systemic limitations, future translational initiatives should emphasize site-specific vascular delivery systems, such as VSMC-homing biomimetic nanoparticles functionalized with targeting ligands for synthetic VSMCs, alongside localized catheter-based interventions utilizing STAT3 inhibitor-coated drug-eluting stents or balloons. Integrating precision delivery platforms with biomarker-guided patient stratification will facilitate the transition of STAT3-targeted therapeutics from broad-spectrum pathway blockade to precise, localized microenvironmental modulation, ultimately establishing safer and more efficacious therapeutic paradigms for vascular remodeling diseases.

## Figures and Tables

**Table 4:** Translational and clinical STAT3 inhibitors.

Therapeutic Agent	Target/Mechanism of Action	Development Stage/Clinical Identifier	Key Clinical Findings & Cardiovascular Relevance	References
Tofacitinib	JAK1/JAK3 inhibitor (indirect STAT3)	FDA-approved; cardiovascular observational cohorts	Improves endothelium-dependent vasodilation, halts carotid intima-media thickening, and suppresses pro-atherogenic cytokines.	[82,277,278]
Ruxolitinib	JAK1/JAK2 inhibitor (indirect STAT3)	FDA-approved; preclinical vascular translation	Suppresses post-angioplasty neointimal formation; attenuates vascular smooth muscle cell dedifferentiation and inflammatory signaling.	[60,272,274]
TTI-101 (C188-9)	Direct STAT3 SH2 domain inhibitor	Phase 1/2 (NCT03195699)	Confirms oral bioavailability, safety profile, and on-target STAT3 engagement in humans; suppresses pathological tissue remodeling.	[253]
Ziltivekimab	Anti-IL-6 ligand mAb (upstream STAT3)	Phase 2 (RESCUE)/Phase 3 (ZEUS, NCT05021835)	Markedly reduces hsCRP, fibrinogen, and vascular inflammatory burden in high-risk atherosclerotic patients.	[311,312]
Tocilizumab	Anti-IL-6 receptor mAb (upstream STAT3)	Phase 2 (ASSAIL-MI/NSTEMI trials)	Reduces systemic vascular inflammation, improves arterial compliance, and attenuates myocardial ischemic injury post-PCI.	[313,314]
Danvatirsen (AZD9150)	STAT3 Antisense Oligonucleotide (ASO)	Phase 1/2 (NCT02983578, NCT05986240)	Selectively downregulates STAT3 mRNA expression; demonstrates clinical feasibility of direct genetic STAT3 inhibition.	[315,316]

## Data Availability

No new data were created or analyzed in this study. Data sharing is not applicable to this article.

## Abbreviations

The following abbreviations are used in this manuscript:

STAT3	Signal transducer and activator of transcription 3
VSMCs	Vascular smooth muscle cells
CVDs	Cardiovascular diseases
ECM	Extracellular matrix
NTD	N-terminal domain
CCD	Coiled-coil domain
DBD	DNA-binding domain
iNOS	inducible nitric oxide synthase
LD	Linker domain
SH2	Src homology 2 domain
PDGF	Platelet-derived growth factor
EGF	Epidermal growth factor
JAKs	The Janus kinases
MAPKs	Mitogen-activated protein kinases
mTOR	mammalian target of rapamycin
PKCδ	Protein kinase Cδ
PAR-1	Protease-activated receptor 1
GSK-3α/β	Glycogen synthase kinase 3α/β
CBP	CREB-binding protein
SOCS	Suppressors of cytokine signaling
PIAS	Protein inhibitors of activated STAT
PTPs	Protein tyrosine phosphatases
KIR	Kinase inhibitory region
PTPRD	Receptor-type protein tyrosine phosphatase D
PTPRT	Receptor-type PTP T
PTPRK	Receptor-type PTPK
SHP1	Src homology 2 domain-containing protein tyrosine phosphatase 1
PTPN9	PTP non-receptor type 9
TC-PTP	T-cell PTP
PTPN2	PTP non-receptor type 2
PCNA	Proliferating cell nuclear antigen
HK2	Hexokinase 2
mitoSTAT3	Mitochondrial STAT3
ETC	Electron transport chain
ERSTAT3	Endoplasmic Reticulum STAT3
IP3R3	Inositol 1,4,5-trisphosphate receptor type 3
GRIM-19	Gene associated with retinoid-IFN-induced mortality-19
PKR	Protein kinase R
α-SMA	α-smooth muscle actin
SM22α	Smooth muscle 22α
SM-MHC	Smooth muscle myosin heavy chain
MMPs	Matrix metalloproteinases
GAS	Gamma-interferon-activated sequence
SRF	Serum response factor
TET	Ten-Eleven Translocation
HATs	Histone acetyltransferases
H3K27ac	Histone H3 at lysine 27
Ang II	Angiotensin II
MMP-2	Matrix metalloproteinase-2
MMP-9	Matrix metalloproteinase-9
WDR1	WD repeat domain 1
ADF	Actin-depolymerizing factor
FAK	Focal adhesion kinase
NLRP3	The NOD-like receptor thermal protein domain associated protein 3
AxCAdnSTAT3	An adenoviral vector to construct a dominant-negative STAT3
GSDMD-NT	GSDMD N-terminal domain
GSDME-NT	GSDME N-terminal domain
AD	Aortic dissection
LILRB4	Leukocyte immunoglobulin-like receptor B4
VC	Vascular calcification
Hcy	Hyperhomocysteinemia
PKM2	Pyruvate kinase M2
LDHA	Lactate dehydrogenase A
ROS	Reactive oxygen species
GLS	Glutaminase
α-KG	α-ketoglutarate
TCA	Tricarboxylic acid
ox-LDL	oxidized low-density lipoprotein
PDK1	Pyruvate dehydrogenase kinase 1
HIF-1α	Hypoxia-inducible factor1-α
KLF4	Krüppel-like factor 4
TIMP2	tissue inhibitor of metalloproteinases 2
EGCG	Epigallocatechin-3-gallate
CT	Cryptotanshinone
